# A delivered DNase toxin creates population heterogeneity through transient intoxication of siblings

**DOI:** 10.1128/mbio.02083-25

**Published:** 2025-09-22

**Authors:** Hanna Eriksson, Susan Schlegel, Sanna Koskiniemi

**Affiliations:** 1 Department of Cell and Molecular Biology, Uppsala University 206112, Uppsala, Sweden; University of California, Berkeley, Berkeley, California, USA

**Keywords:** toxin delivery, contact-dependent growth inhibition, bacteria, population heterogeneity, multicellularity, bet-hedging, SOS-response

## Abstract

Population heterogeneity is important for multicellular behavior, as well as bet-hedging strategies. Recent findings suggest a role for bacterial toxin delivery in generating population heterogeneity, but the molecular mechanisms by which this occurs are not well understood. Here, we address if and how delivery of bacterial CdiA toxins generates heterogeneity in an isogenic population of *Escherichia coli* (*E. coli*) cells. Using a DNase toxin as a proxy, we find that *E. coli* populations able to deliver the toxin show a heterogeneous expression of the SOS-response gene *sulA*, whereas those incapable of kin-delivery remain homogeneous. Heterogeneity results from excessive delivery of toxin into some cells, which become intoxicated due to insufficient immunity. A low level of intoxication by this toxin is transiently reversible, and intoxicated cells can be rescued by the *de novo* synthesis of cognate immunity protein. The fraction of cells experiencing toxicity is increased by liberating the receptor responsible for toxin import from its tasks in outer-membrane biogenesis, suggesting that kin-intoxication is limited by receptor availability. Expression of *sulA* is regulated by both DNA damage and redox status. Interestingly, kin-delivery changes redox status, whereas intoxicated non-kin cells induce the SOS DNA damage response. The former results in changed expression of metabolic genes, whereas the latter induces prophage excision, which may promote horizontal gene transfer. In conclusion, we identify a molecular mechanism by which heterogeneity is generated through toxin delivery among kin, and some of the consequences of said heterogeneity.

**IMPORTANCE:**

Population heterogeneity is important for multicellularity, as well as for bet-hedging strategies. A heterogeneous population allows cells with the same genotype to respond differently to environmental cues and stresses. For multicellularity, heterogeneity originates from coordinated signaling, whereas bet-hedging strategies can arise stochastically due to cell-to-cell variation in the concentration of signaling molecules. However, recent advances suggest a role for bacterial toxin delivery in the generation of population heterogeneity. How toxins mediate heterogeneity mechanistically is, however, unclear. Here, we show that kin cells transiently intoxicate each other with CdiA toxins, resulting in physiological changes. These changes are specific to the toxic activity, i.e., other toxins with different activities are likely to give rise to other responses. Thus, we find that the arsenal of toxins that bacteria harbor could affect their ability to participate in bet-hedging strategies, as well as in multicellular behavior.

## INTRODUCTION

Most bacterial species participate in multicellular behavior, such as, e.g., biofilm formation (1). For multicellular behavior, communication is essential. To communicate, bacteria secrete soluble chemicals that allow them to sense the presence of other bacteria of the same species, a phenomenon known as quorum sensing (2). Notably, in “true” multicellular organisms, direct cell-to-cell signaling is found alongside the production of soluble signaling molecules (3). Early work suggested that contact-dependent growth inhibition (CDI), a toxin delivery system in bacteria, could be used for contact-dependent signaling during biofilm formation (4). Although recent findings show that toxin delivery is not relevant in this context (5), accumulating evidence suggests that delivery of bacterial toxins between cells through contact-dependent mechanisms results in population heterogeneity (6, 7). Generation of population heterogeneity is not *per se* a sign of multicellular behavior. Population heterogeneity is also useful as a bet-hedging strategy where short-term fitness is sacrificed in a fraction of the cells to ensure survival of the population upon rapid changes to harsh conditions (8). For example, a subpopulation of transiently non-growing cells is able to survive antibiotic exposure without the accumulation of resistance mutations, a phenomenon known as persistence (9). Thus, population heterogeneity mediated by bacterial toxin delivery systems could be important for bet-hedging strategies or multicellular behavior. However, if and how bacterial toxins mediate population heterogeneity and gene-expression changes on the molecular level is still not entirely clear.

Toxin-mediated heterogeneity in gene expression could arise by at least two distinct molecular mechanisms: (i) the toxins, either by themselves or in concert with the immunity protein, could act as transcriptional regulators, as has been seen with type II TA-systems (10, 11) or (ii) the toxins could change gene expression through their toxicity, as shown for, e.g., type II toxins that specifically cleave certain mRNA (12). Both the toxin and immunity of these TA systems are expressed inside the cell, and when the toxin:antitoxin balance is ever disrupted is still unclear for many systems. For antibacterial toxins delivered between kin-bacteria, such as type 6 secretion system effectors, bacteriocins, or CdiA toxins, the ability to change the ratio between toxin and antitoxin is, on the other hand, built into the system. Delivery of a toxin to a kin cell (a cell with antitoxin) will increase the number of toxins in the receiving cell, changing the toxin:antitoxin ratio and possibly affecting gene expression through either of the mechanisms described above.

**Fig 1:**
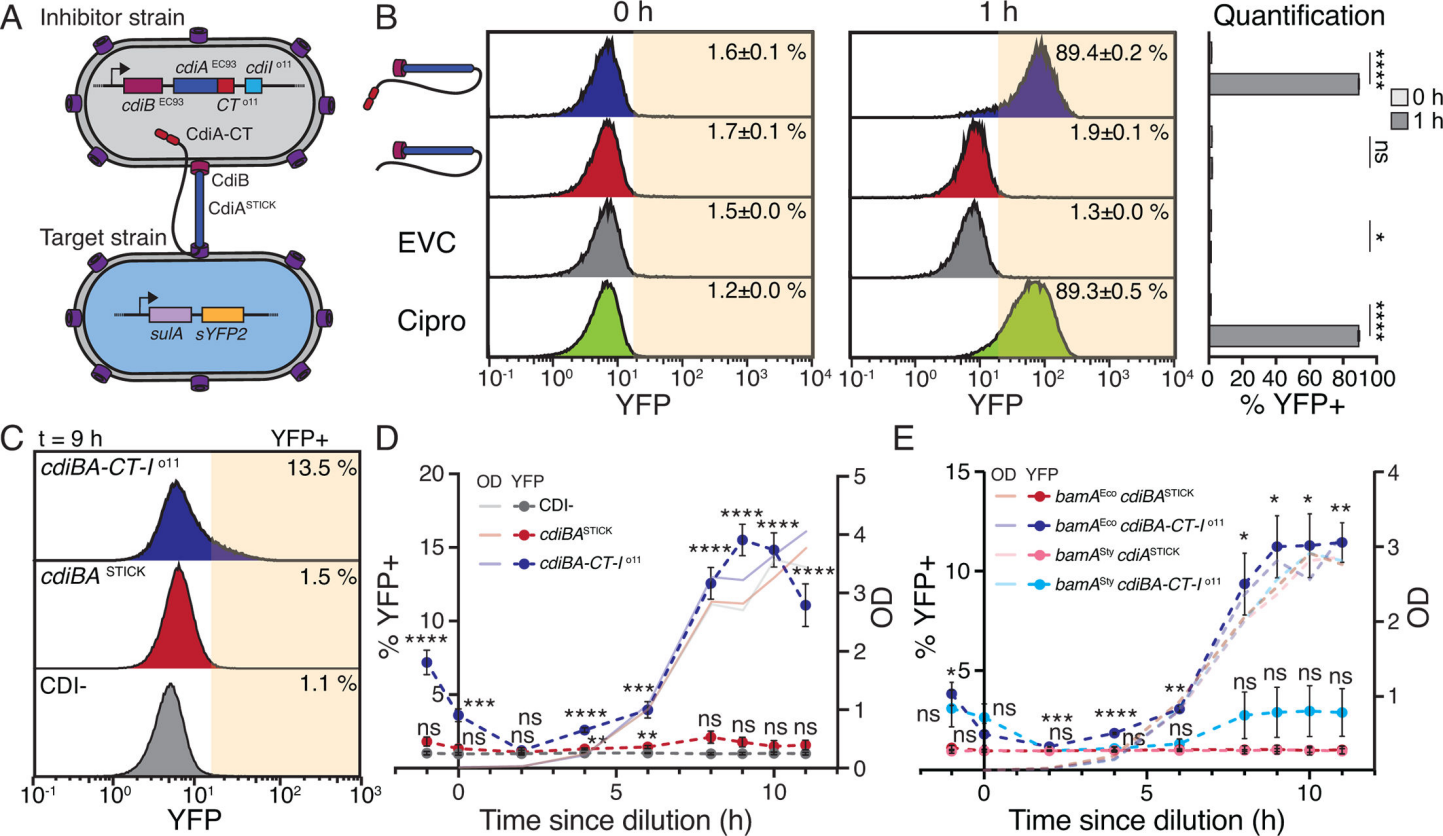
CDI*+ E. coli* intoxicate their siblings during monoculture. (**A**) Overview of strains used for competition in B. (**B**) Competition between MG1655 inhibitor cells with or without p*cdiBA-CT-I*^o11^ and MG1655 target cells with *sulA-sYFP2* reporter. Enumeration of YFP+/− cells through flow cytometry, as shown in histograms and in a bar chart. Ciprofloxacin (Cipro) was used as a control. t0 = before mixing, t1 = after 1 h of co-culture. EVC = empty vector control. *N* = 6. (**C**) Representative distribution of YFP+ events in a monoculture of MG1655 with or without p*cdiBA-CT-I*^o11^ or p*cdiBA*^STICK^. The yellow area indicates the YFP+ population, which is determined as the area where CDI^−^ cells have approximately 1% YFP+ events in each experiment. (**D and E**) Time-resolved enumeration of YFP+ cells in monocultures grown in M9-gly-CAA for 11 h. MG1655 with p*cdiBA-CT-I*^o11^ (blue) or p*cdiBA*^STICK^ (red) was used in (**D**), and MG1655 with p*cdiBA-CT-I*^o11^ or p*cdiBA*^STICK^, either with cognate *bamA*^Eco^ (red and blue) or non-cognate *bamA*^Sty^ (pink and light blue), was used in (**E**). *N* = 12 (**D**), 4 (**E**). CDI− (gray) is MG1655 with the *sulA-sYFP2* reporter. Error bars are SEM. Statistical significance was determined through (**B**) Student’s *t*-test and (**D and E**) two-way analysis of variance (ANOVA) with Tukey’s post hoc test. * <0.05, ** <0.01, *** <0.001, **** <0.0001. Significance in D, E is relative to the respective *cdiBA*^STICK^ strain.

One example of toxin delivery that requires direct cell-cell contact is CDI (13). CDI systems are encoded by a three-gene locus *cdiBAI* and are found in many Gram-negative bacteria, including some *Escherichia coli* strains. During CDI, cells with the systems use the CdiB beta-barrel exporter to present the stick-like CdiA toxin on their cell surface. Upon direct interaction with a target cell expressing the cognate outer-membrane receptor (e.g., BamA), CdiA is proteolytically processed at a conserved VENN motif, resulting in transfer of the C-terminal toxin domain (CdiA-CT) into the target cell periplasm (Fig. 1A) (14). To access the cytosol or inner membrane, where most characterized CdiA toxins exert their function, the N-terminal part of the CT (also known as the C-terminal entry domain, or CED) interacts with a cognate protein in the inner membrane that facilitates cytosol or membrane entry in an as-yet undefined manner (15). CdiA-CTs are polymorphic toxins with different biological activities, including ionophores and nucleases with DNase or RNase activity (16, 17). To avoid self-inhibition, cells produce a CdiI immunity protein that protects against its corresponding CdiA-CT toxin (18).

Here, we set out to investigate if and by what mechanism a CdiA toxin causes population heterogeneity. We chose a toxin with DNase activity as a proxy, as this allows identification of intoxication using an established fluorescent reporter, and used the laboratory strain *E. coli* MG1655 for the study. We find that a subpopulation of cells expressing this CDI system becomes intoxicated during dense growth in M9 minimal media. Intoxication is delivery-dependent and limited by receptor availability and appears to occur when the toxin molecules outnumber the antitoxins, rather than being a consequence of antitoxin instability. Intoxicated cells can resume growth after *de novo* synthesis of immunity protein to a certain level of intoxication, indicating a reversible process. These cells change their gene expression, but the changes depend on the degree of intoxication. During kin delivery, slight intoxication results in altered expression of metabolic genes, whereas increased toxicity also induces the SOS DNA damage response, resulting in cell death and prophage excision. Taken together, our results suggest that kin-delivery of toxins could function as a contact-dependent signaling mechanism by which the redox status and gene-expression pattern of a subpopulation of cells is changed at high population densities.

## RESULTS

### Delivery of CdiA-CT^o11^ toxin among kin induces *sulA* expression in a subpopulation

To investigate if kin-delivery of a DNase toxin could generate population heterogeneity, we used a previously described yellow fluorescent protein (YFP) based transcriptional reporter, *sulA-sYFP2,* that signals when toxicity occurs in an individual cell. DNA damage induces the LexA-controlled SOS DNA damage response genes in *E. coli*, including the cell-division inhibitor protein SulA (19). To investigate if delivery of the CdiA-CT^o11^ toxin (with known DNase activity [17]) induced *sulA-sYFP2*, we competed *E. coli* with plasmid-encoded p*cdiBA-CT-I*^o11^ locus (inhibitor cells) against target cells with the *sulA-sYFP2* reporter. Target cells constitutively expressed blue fluorescent protein (BFP) and inhibitor cells red fluorescent protein (dTomato) to enable separation of the two (Fig. 1A). Using flow cytometry, we found that 89% of the target cells became YFP-fluorescent after 1 h of co-culture with inhibitor cells, indistinguishable from the ciprofloxacin-treated positive control (Fig. 1B). In contrast, less than 2% YFP+ cells were observed when inhibitor cells carried either an empty vector control devoid of CDI (EVC) or an empty stick control (p*cdiBA*^STICK^) lacking *cdiA-CT-I*^o11^ (Fig. 1B). In agreement with the fluorescence data, target cells competed with control strains (EVC, p*cdiBA*^STICK^) survived well (monitored by viable counts on selective agar plates), whereas survival dropped to 2% for target cells competed with inhibitor cells carrying the CdiA-CT^o11^ toxin (Fig. S1A). This suggests that the *sulA-sYFP2* reporter is a functional readout for CdiA-CT^o11^ intoxication. However, while intoxication prevented growth of 98% of the cells, *sulA-sYFP2* was only induced in 89% of the population, suggesting that excessive damage prevents some of the intoxicated cells from inducing the DNA damage response. To test if this was the case, we cloned CdiA-CT^o11^ under an arabinose-inducible promoter in cells with a chromosomal *cdiBA-CT-I*^o11^ locus (to mitigate toxicity from leaky expression) and the *sulA-sYFP2* reporter. After overexpression of the toxin for 5 and 20 min, we measured if the percentage of YFP+ cells differed from the percentage of dead cells in the population. Cell death was assessed as the reduction in colony-forming units, and the percentage of dead cells was calculated as the loss in viability during induction, relative to the starting cfu/mL [%Death = (cfu/mL_t0_ − cfu/mL_tX_)/cfu/mL_t0_]. After 20 min, 90% of the cells were dead, which is significantly higher than the 60% YFP+ cells at the same time point (Fig. S1B), suggesting that excessive damage due to over-intoxication prevents detectable *sulA-sYFP2* induction in some cells.

As the majority of CDI systems are found in a single copy on the bacterial chromosome (20), we incorporated the *cdiBA-CT-I*^o11^ or *cdiBA*^STICK^ locus into the chromosome to investigate the consequences of kin delivery. Cells carrying chromosomal *cdiBA-CT-I*^o11^ induced the SOS response in around 77% of sensitive target cells and inhibited growth (Fig. S1C and D), confirming functional toxin delivery. To investigate if kin-delivery of CdiA-CT^o11^ can cause population heterogeneity, *sulA-sYFP2*-containing cells with chromosomal *cdiBA-CT-I*^o11^ or *cdiBA*^STICK^ were grown in monoculture, and the percentage of YFP+ cells in the population over time was monitored using flow cytometry. The YFP threshold was set for each time point as the value where the CDI^−^ control had an average of 1% cells above the threshold (cells above the threshold are termed YFP+) (Fig. 1C). Heterogeneity was assessed as a change in YFP signal distribution in the bacterial population. Cells expressing *cdiBA-CT-I*^o11^ had significantly different distribution in YFP signal compared to *cdiBA*^STICK^ or empty vector control (Fig. 1C) (Chi-square test *P* < 0.0001). At higher cell densities (OD_600_ ≈ 3, Fig. 1D), *sulA-sYFP2* was induced in >10% of the cells expressing *cdiBA-CT-I*^o11^, while no increase in fluorescence was observed for the population with *cdiBA*^STICK^ (Fig. 1D). When delivery was restricted by replacing the native, cognate receptor *bamA*^Eco^ by a non-cognate variant from *Salmonella enterica* serovar Typhimurium LT2, *bamA*^Sty^ (21), no increase in the % of YFP+ cells was detected (Fig. 1E). Together, these results suggest that kin-delivery of CdiA-CT^o11^ can cause heterogeneity in *sulA* expression.

### Intoxication of cells by toxin over-delivery

The intoxication of only a subpopulation of cells raises questions regarding the molecular mechanism at play, i.e., what makes some cells in an isogenic population sense the toxin while their siblings do not? A reasonable assumption is that the toxins outnumber the immunity proteins in the affected cells.

To investigate whether variations in the toxin:immunity ratio could arise from differences in toxin or immunity protein turnover rates, the stability of CdiA-CT^o11^ and CdiI^o11^ was assessed by western blot following DL-serine hydroxamate-mediated translation arrest. Whereas CdiI^o11^ appears completely stable even 120 min after translation arrest (Fig. S2A and B), CdiA-CT^o11^ with a C-terminal alpha tag (CdiA-CT^o11^-α) was degraded below the detection limit at the same time point (Fig. S2C). This suggests that the observed heterogeneity is unlikely to be due to proteolytic turnover of the immunity protein.

**Fig 2:**
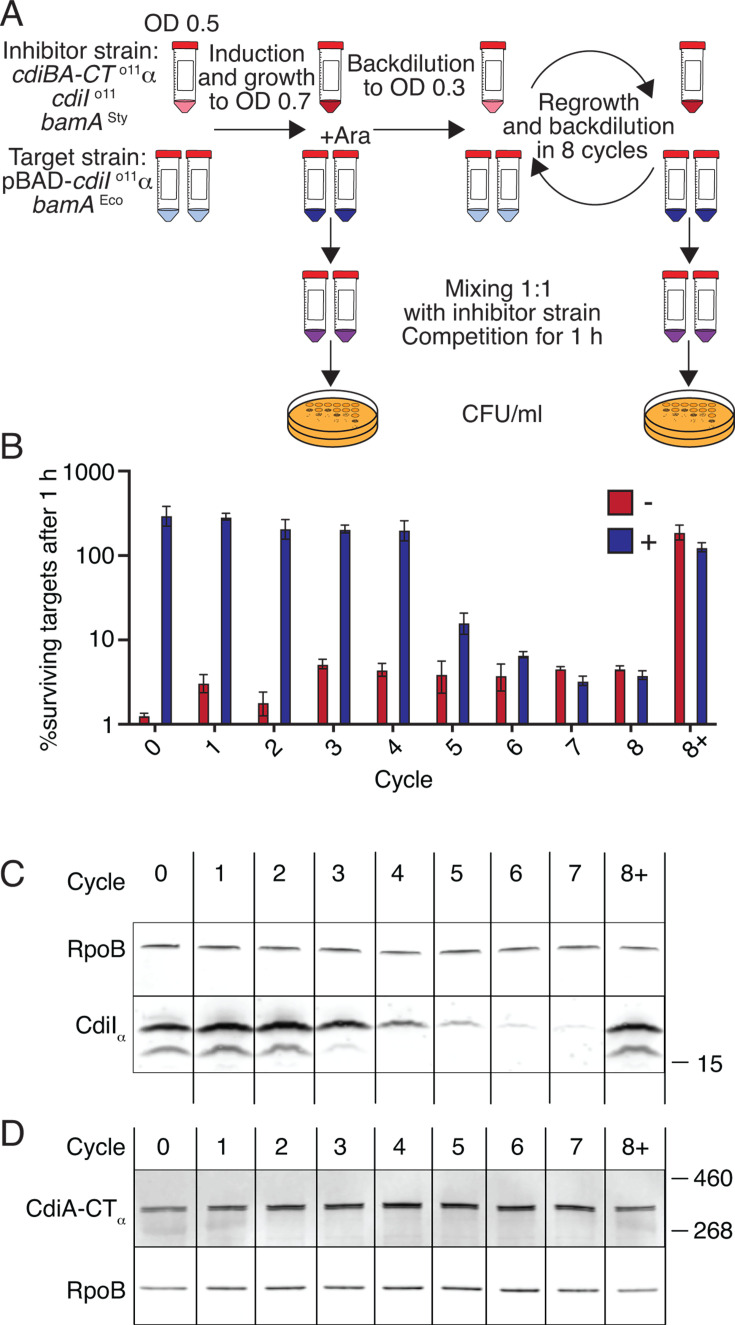
Target cells become intoxicated when toxin levels outnumber immunity levels. (**A**) Overview of the experiment. (**B**) Competitive index after co-culturing p*cdiBA-CT-*α*-I*^o11^ inhibitor cells with target cells containing different levels of CdiI^o11^-α immunity (as seen in C) for 1 h. Red/blue bars indicate target cells grown in the absence/presence of L-arabinose before cycle 0. Error bars show SEM, *N* = 3. (**C**) Western blot to detect CdiI^o11^-α immunity in target cells using anti-α nanobodies. (**D**) Western blot to detect the level of full-length CdiA^o11^-α in inhibitor cells (expressed by the inhibitor cells throughout the different cycles of the experiment) using anti-α nanobodies. To assess uniformity in sample loading, *E. coli* RNA polymerase beta subunit (RpoB) was detected using an anti-RpoB antibody (**C, D**). Samples for cycle 8 were omitted from the western blot due to the size limitation of the gel.

To assess if kin-intoxication instead could originate from an over-delivery of toxin relative to the protective capacity of individual cells, we tested if a gradual decrease in immunity would result in intoxication upon constant toxin delivery. To modulate levels of immunity, target cells were supplemented with a plasmid expressing arabinose-inducible *cdiI*^o11^-α (p*cdiI*^o11^-α). The ALFA-tag was added to the immunity to be able to discriminate it from CdiI expressed in the inhibitor cell population (Fig. 2A) and did not change the stability of the protein (Fig. S2D). The population level of CdiI^o11^-α was altered using a method inspired by the fluorescence dilution assay (22). Exponentially growing target cells were either left uninduced (−) or induced (+) with 0.02% L-arabinose for 15 min to obtain a homogeneous expression of *cdiI*^o11^-α in individual cells (expression of *cdiI*^o11^-α-*mTagBFP* is bimodal at concentrations lower than 0.002% [Fig. S2A and B]). Subsequently, cultures were diluted and re-grown in eight 2 h cycles, equating to ~19 generations in total (Fig. 2A). Assuming that degradation is negligible (Fig. S2D), the amount of CdiI^o11^-α should decrease by half at every cell division, resulting in an approximately fivefold dilution during each 2 h cycle (Fig. S3D). At the end of each cycle, samples were taken for (i) a competition against inhibitor cells with p*cdiBA-CT*-α*-I*^o11^ (Fig. 2B) and (ii) western blotting to monitor levels of immunity in the target cells and levels of full-length CdiI-CT^o11^-α in the inhibitor cells (Fig. 2C and D). While toxin levels remained constant over time (Fig. 2D), immunity levels decreased with the anticipated approximately fivefold dilution per cycle after cycle 2 (Fig. 2C; Fig. S3D). Target cells showed decreasing protection after cycle 5, when they were outcompeted 10-fold (Fig. 2B, blue bars). At later cycles, diluted (+) target cells were outcompeted to the same extent as non-induced control cells (red bars, Fig. 2B). To check that the target cells had maintained the immunity plasmid, the cultures were split at cycle 8, and expression of immunity was re-induced in ½ of each culture irrespective of previous induction status (cultures termed 8+). Cells from both +/− cultures showed full protection against CdiA-CT^o11^ (Fig. 2B) and the levels of CdiI^o11^-αwere restored (shown for culture 8+ in Fig. 2C). Taken together, these results suggest that growth is inhibited when toxin delivery exceeds the capacity of the available immunity proteins.

### Intoxication by CdiA-CT^o11^ is reversible within a short time window

**Fig 3:**
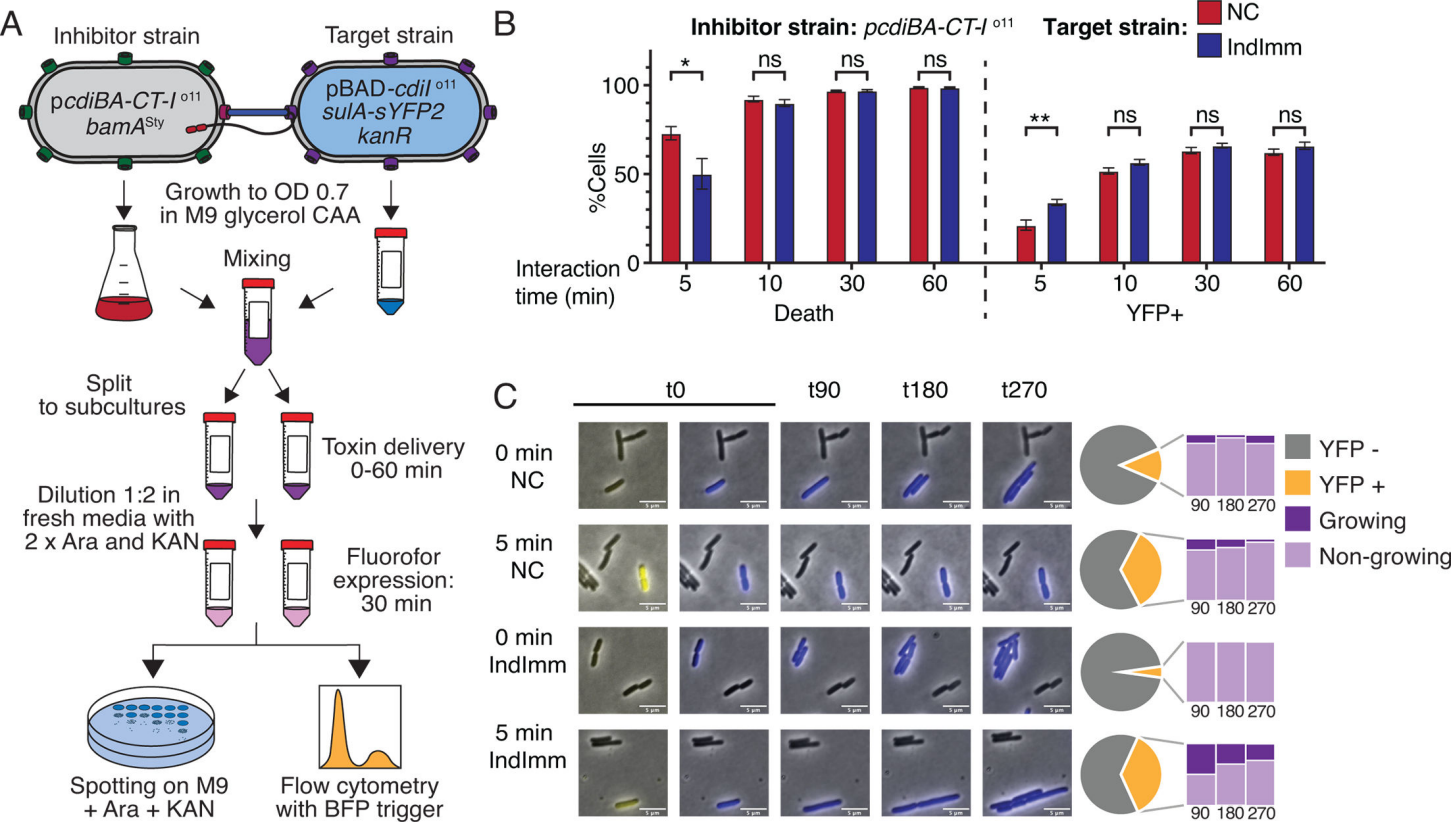
Cells intoxicated by CdiA-CT^o11^ resume growth upon induction of *cdiI*^o11^. (**A**) Overview of the experiment. (**B**) % Dead and % YFP+ target cells with (IndImm, blue bars) or without (NC, red bars) pBAD::*cdiI*^o11^ after co-culture with p*cdiBA-CT-I*^o11^ inhibitor cells for 5, 10, 30, or 60 min. *N* = 8. Error bars are SEM. Statistical significance was determined using Student’s *t*-test. * >0.05, ** >0.01. (**C**) Time-lapse microscopy on cells from the competition in B. Cells are assessed as YFP+ (yellow) or YFP− (gray). The fraction of YFP+ target cells that do (dark purple) or do not (light purple) resume growth after 90, 180, and 270 min of recovery is shown. *N* = 66, 53, 82, and 109 for t0 IndImm, t0 NC, t5 IndImm, and t5 NC samples, respectively.

For an intoxication event to result in a response other than growth inhibition, the damage caused by the toxin must be reversible. To investigate whether CdiA-CT^o11^-intoxicated cells can be rescued, target cells with either an arabinose-inducible *cdiI* plasmid (IndImm) or an empty vector control (NC) were competed with p*cdiBA-CT-I*^o11^ inhibitor cells. Arabinose was added to the cultures 0, 5, 10, 30, or 60 min after mixing (Fig. 3A). The cells were left to recover for 30 min before fluorescence and cell death were assessed in the total target cell population (as described for Fig. S1). When immunity was induced after 5 min of competition, 50% of the target cells died compared to 75% of the non-immune target cells (NC) (Fig. 3B, left panel). 34% of the immunity-induced target cells were YFP+, compared to 20% in the NC (Fig. 3B, right panel). After 10 min of competition, 90% to 95% of target cells died, and YFP+ cells increased to ~50% regardless of immunity status (Fig. 3B). Thus, immunity induction was able to reverse intoxication, but only for a short time, as more target cells survived when immunity was induced after 5 min, but not at 10 min. The observation that the % of dead cells exceeds the % of YFP+ cells at either time point agrees with our previous observation that many affected cells die before being able to induce the DNA damage response (Fig. S1B).

Even though our results indicate that DNase-mediated intoxication can be reversed—if only for a short time—bulk experiments do not reveal which cells in the population grow. We therefore repeated the assay described in Fig. 3A but conducted the recovery phase on agarose pads and followed the growth resumption of individual cells by time-lapse microscopy. Both p*cdiI*^o11^ and non-immune cells resumed growth when recovery was initiated immediately after mixing with inhibitor cells (0 min). Few cells showed signs of *sulA-sYFP2* expression (Fig. 3C; Fig. S4A), indicating that negligible amounts of toxin had been delivered. After 5 min of competition, the fraction of YFP+ cells had increased similarly in both target strains, but after 90 min of recovery, the fraction of YFP+ target cells that could resume growth was higher in p*cdiI*^o11^ cells (50%) than in the empty vector control (18%) (Fig. 3C; Fig. S4B and C). At later time points, the number of cells that resumed growth decreased for both target strains, possibly because some cells lysed after resuming growth. Nonetheless, the fraction that could resume growth was larger for p*cdiI*^o11^ cells than for the control (Fig. 3C). These results show that increasing levels of immunity can mitigate the effects of intoxication by CdiA-CT^o11^ in part of the population.

### CdiA-CT import depends on the number of outer membrane receptors

**Fig 4:**
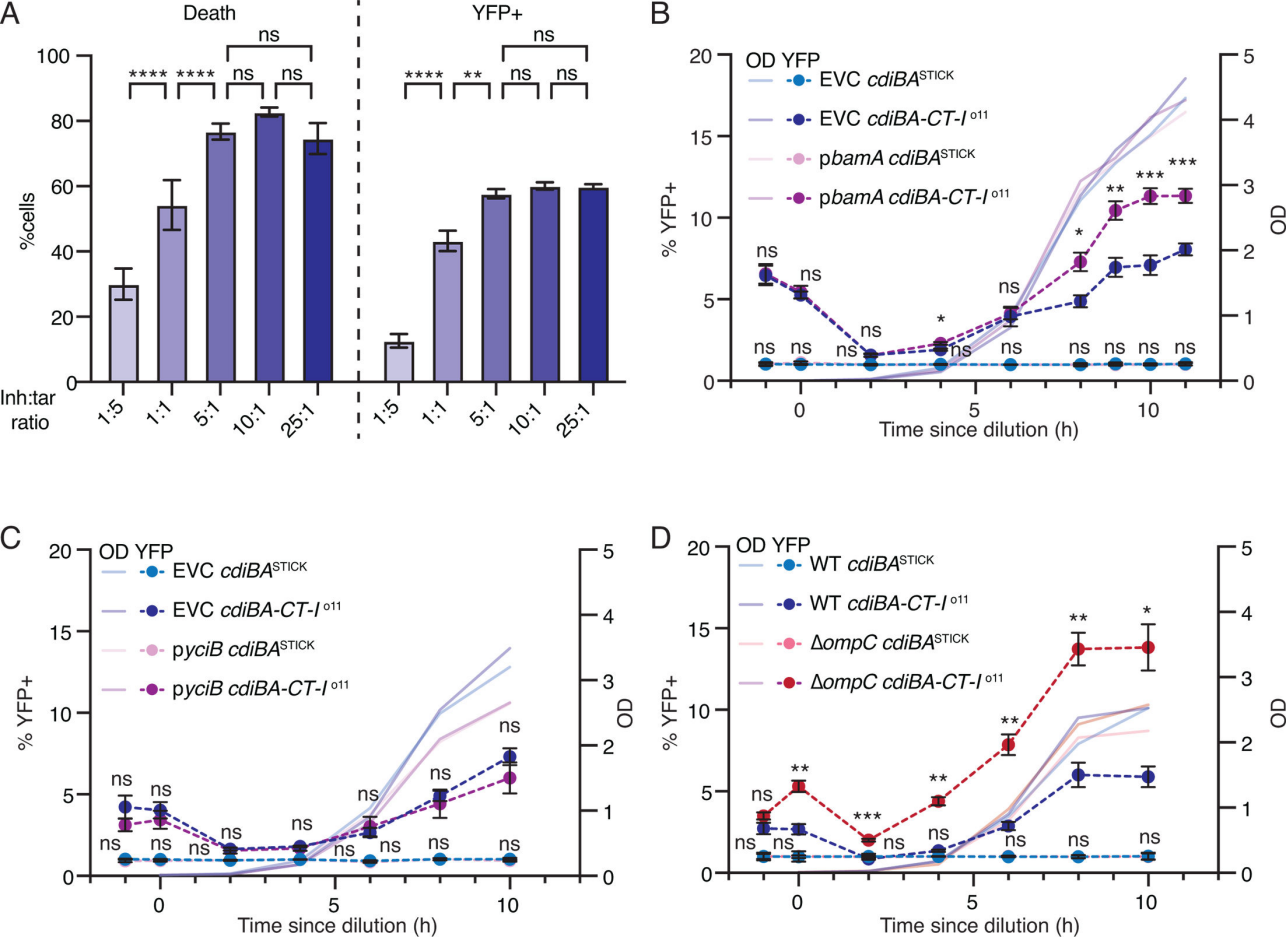
BamA levels restrict toxin import. (**A**) % Dead and YFP+ target cells after 5 min of co-culture with p*cdiBA-CT-I*^o11^ inhibitor cells mixed at increasing inhibitor:target cell ratios. *N* = 6. (**B–D**) Time-resolved enumeration of YFP+ cells and cell density (OD_600_) in monocultures grown in M9-gly-CAA. Cells in monocultures: MG1655 with chromosomal *cdiBA-CT-I*^o11^ or *cdiBA*^STICK^ and an empty vector (EVC [**B, C, D**]), p*bamA* (**B**), p*yciB* (**C**), or Δ*ompC* (**D**). Average OD_600_ values for the strains are shown as shaded lines. *N* = 6, 9, 4 (**B, C, D**). Error bars are SEM. Statistical significance was determined through two-way ANOVA with Tukey’s post hoc test. * <0.05, ** <0.01, *** <0.001, **** <0.0001.

As shown above, some cells in an isogenic culture become intoxicated by CdiA-CT^o11^ because too much toxin is delivered to these cells. This raises the question of why more toxin is delivered to some cells than others. A possible explanation is that some cells receive more toxin simply because they meet more inhibitor cells. In that case, increasing the ratio of inhibitor:target cells should result in increased inhibition. This was investigated by varying the inhibitor:target cell ratio from 1:5 to 25:1, keeping the time for competition constant (5 min) and using only non-immune target cells. Indeed, more target cells died with an increased fraction of inhibitor cells until a ratio of 5:1 (Fig. 4A). However, no further increase in death or in the percentage of YFP+ cells could be observed at a 10:1 or 25:1 ratio (Fig. 4A), suggesting that cell-cell contacts and toxin delivery are saturated at a 5:1 ratio in a 5 min time frame. However, the fraction of dead target cells increases with time even at this saturating ratio (compare 81% death after 5 min to 99% death after 60 min) (Fig. 3B), suggesting that another factor must limit the number of toxins that can be delivered to any cell during a given time. A possible explanation could be that the number of available import proteins in the target cell limits toxin delivery.

For CdiA-CT^o11^, two proteins are important for toxin import: the outer-membrane receptor BamA and the inner-membrane helper protein YciB (23). To test whether the abundance of these proteins was limiting for toxin import, *bamA* and *yciB* were cloned under a leaky pTet promoter, and the competitions from Fig. 3B were repeated with target cells carrying an empty, p*bamA*, or a p*bamA-yciB* vector. Target cells expressing p*bamA* showed more death compared to target cells with empty vector control, already 5 min after mixing with inhibitor cells, and this continued to increase throughout the time course (Fig. S5A and B). No increase in YFP+ cells could be observed (Fig. S5C and D), in line with our previous observation that too much toxin results in cell death without SOS induction. Target cells with p*bamA-yciB* showed a similar percentage of survival and YFP levels as target cells with p*bamA* (Fig. S5), indicating that *bamA* and not *yciB* levels limit intoxication.

The number of outer membrane proteins is likely to vary between individual cells due to fluctuating gene expression according to normal distribution (24). If the number of receptor proteins dictates the rate of toxin import, this could also lead to variation in the number of toxins delivered between individual cells in an overnight culture, providing a potential explanation for why only some cells sense the toxin in an isogenic population. To test this, we repeated the monoculture experiment with cells expressing either *bamA* (Fig. 4B) or *yciB* (Fig. 4C). In agreement with the previous results, cells provided with additional *bamA* showed an increased fraction of YFP+ cells as compared to the empty vector control (Fig. 4B). No such increase was observed when cells expressed *yciB* (Fig. 4C), and supplying both together did not increase the percentage of YFP+ cells compared to *bamA* alone (Fig. 4B; Fig. S5E). This further supports that the levels of BamA, and not YciB, determine the level of toxin import and that BamA levels limit toxin-delivery-induced population heterogeneity. This finding is rather surprising since the levels of BamA in minimal media (~1,500 molecules per cell [25]) would be expected to promote ample delivery of toxin into any cell. This can be compared to ~200 molecules YciB/cell (25), which does not seem to be limiting for toxin import. A possible explanation is that only a subset of the many BamA molecules is able to participate in toxin import at any given time. BamA is an essential component of the beta-barrel assembly machine, responsible for translocating proteins into and across the outer membrane. In this process, the lateral gate within BamA opens and closes to allow protein translocation. Recent evidence suggests that CdiA proteins interact not only with the outer membrane loops of BamA but also with residues involved in opening of the lateral gate (26). Thus, the majority of BamA proteins, e.g., those currently occupied in secreting proteins or found in a conformation where the loops are inaccessible for CdiA binding (27), may not be available for toxin import. With this in mind, we reasoned that reducing the load on the beta-barrel assembly machinery might liberate BamA proteins for CdiA binding. To test this, we deleted the gene encoding the most abundant beta-barrel protein, OmpC (~100,000 molecules/cell), from the MG1655 genome and repeated the monoculture assay in these cells. Deletion of *ompC* increased the number of YFP+ cells in the monoculture to 14% compared to 6% observed for the wild-type MG1655 in the same experiment (Fig. 4D). Thus, one possibility is that some cells receive too many toxins simply because the number of import-competent outer-membrane receptors is high enough to allow toxin uptake at a level that cannot be neutralized by the cognate immunity proteins in the same cell. However, it is also possible that the increased availability of BamA affects the cell in some other way that increases sensitivity to the toxin, for example, by affecting the metabolism of the cells rather than toxin import.

### CdiA intoxication changes cellular metabolism and redox status

**Fig 5:**
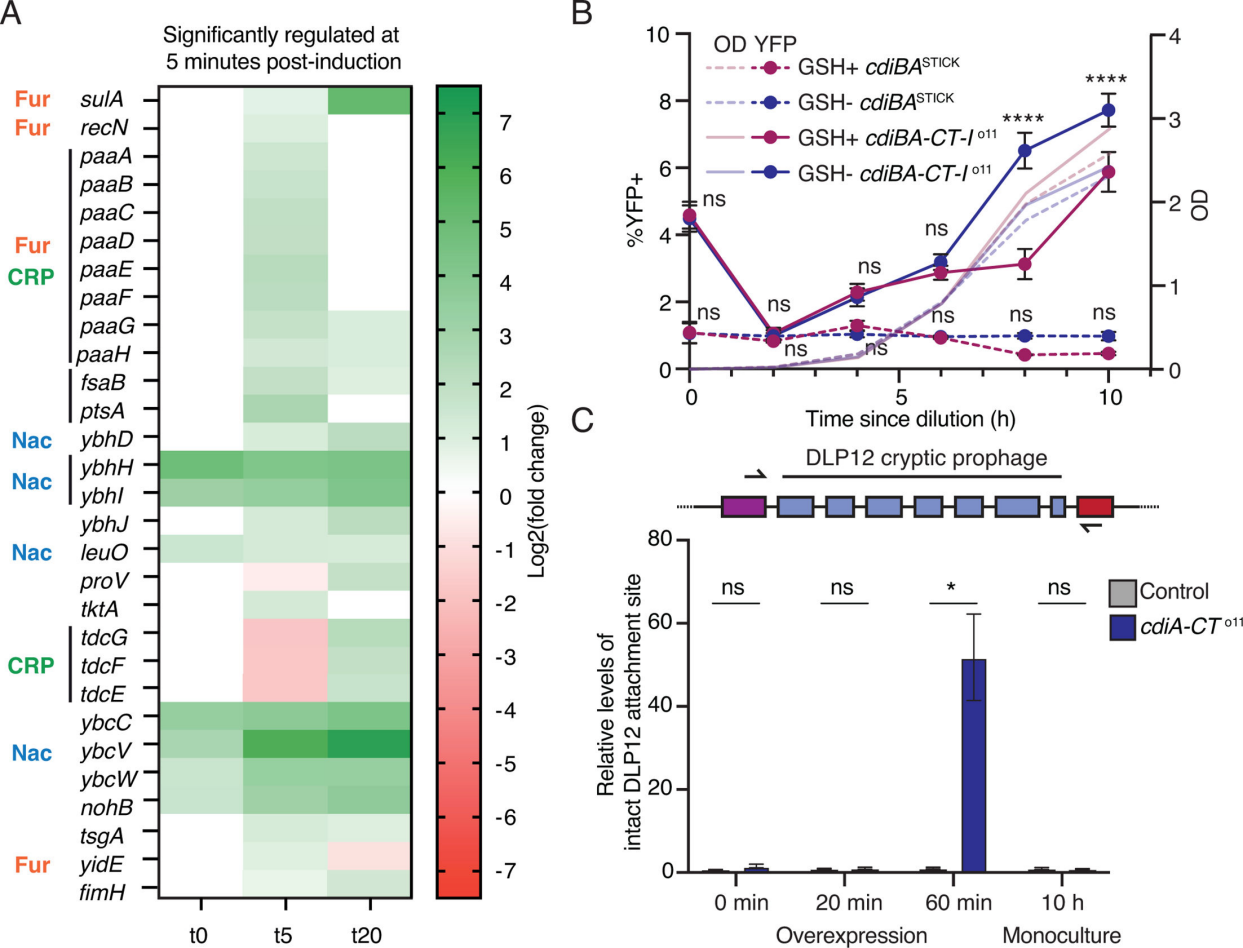
Phenotypic responses to CdiA-CT^o11^ intoxication vary between kin- and non-kin cells. (**A**) Heatmap of all genes with altered mRNA levels 5 min post-induction of pBAD-*cdiA-CT*^o11^ as compared to empty vector control (*P*-adj < 0.01, *N* = 3 biological replicates). Known metabolic regulators of more than one of the included genes (according to EcoCyc) are indicated. Changes are shown as Log2 fold. (**B**) Time-resolved enumeration of YFP+ cells in monocultures of MG1655 cells with either chromosomal *cdiBA-CT-I*^o11^ (solid lines) or *cdiBA*^STICK^ (dashed lines) grown in M9-gly-CAA with (pink) or without (blue) 5 mM glutathione (GSH) for 10 h (*N* = 12 biological replicates). Gates for YFP+ were set for the *cdiBA*^STICK^ cells without GSH for the respective time point. Statistical significance between GSH+ and GSH− for the same genotype was determined. (**C**) Relative levels of an intact DLP12 attachment site as compared to *purA* ORF in genomes of *E. coli* MG1655 were determined either upon arabinose-induced overexpression of *cdiA-CT*^o11^ (0, 20, 60 min) (control: pBAD33^empty^) or after 10 h growth in monocultures with *chromosomal cdiBA-CT-I*^o11^ (control: *cdiBA*^STICK^). DLP12 excision was quantified using quantitative PCR (qPCR) with primers flanking the integration site, where a higher presence of an intact locus indicates excision of the prophage (*N* = 4 biological replicates). Error bars are SEM. Statistical significance was determined using two-way ANOVA with Tukey’s post hoc test for (**B**) and Student’s *t*-test for (**C**), where * <0.05, ** <0.01, *** <0.001, **** <0.0001.

Our experiments demonstrate that kin-intoxication by CdiA toxins can induce population heterogeneity in terms of gene expression, but do not shed light on the evolutionary purpose of such heterogeneity. We reasoned that a more comprehensive understanding of what happens in CdiA-CT^o11^-intoxicated cells could provide valuable leads. Therefore, we used the p*cdiA-CT*^o11^ overexpression plasmid in cells with a chromosomal *cdiBA-CT-I*^o11^ locus (Fig. S1B) and monitored mRNA levels after 0, 5, or 20 min of arabinose induction using transcriptomics. Differential expression was determined relative to an EVC strain (without the *cdiBAI* operon on the chromosome) at each time point. Expression of CdiA-CT^o11^ reduced the viability of the cells, with 1-log after 5 min of induction and 3-logs after 20 min of induction (Fig. S6A). Five minutes after CdiA-CT^o11^ induction, we observed a moderate increase in mRNA levels for a small subset of genes involved in, e.g., the SOS response (*sulA, recN*), phenylacetyl metabolism (*paaA-H*), and prophages (*exoD, ybcVW, nohB*) (Fig. 5A). Similarly, only a few loci showed a decrease in mRNA levels, e.g., *tdcEFG* and *proV* (Fig. 5A). Keeping in mind that the average half-life of mRNAs in *E. coli* is estimated to be 3–8 min (28), it is not surprising that we did not see a stronger decrease at this time point. Genes affected by either the addition of arabinose (*araBAD, araE, araC, ygeA*) or the chromosomal *cdiBAI-*locus (*lacA*) were not considered further.

After 20 min, more than 2,000 genes showed differential mRNA levels compared to the empty vector control (Table S1). To assess which of the time points best represents the physiological state of the YFP-positive cells in the monoculture (compare Fig. 1), we used a previously described reporter for the DNA damage inducible gene *tisB* (19). At 20 min, *tisB*-mRNA levels were altered similarly to *sulA* (42- and 35-fold increase, respectively), but in contrast to *sulA*, no increase in mRNA levels could be observed at 5 min (Fig. S6B). Despite being induced by the delivery of CdiA-CT^o11^ in a competition (Fig. S6C), no *tisB-sYFP2* positive cells could be observed in monoculture (Fig. S6D), suggesting that the 5 min time point best represents the level of intoxication observed in a monoculture.

Unexpectedly, a majority of the genes with differential expression at 5 min are not known to be directly involved in DNA-damage repair. Instead, gene ontology (GO) enrichment analysis using Panther (29) (https://geneontology.org) suggested an over-representation of genes involved in various metabolic processes, including phenylacetate catabolism (>40-fold), fatty acid oxidation (>13-fold), and carboxylic acid catabolism (>4-fold) (Table S1). The expression of genes with altered mRNA levels at 5 min is controlled by a diverse set of global transcriptional regulators, including Nac, Fur, and CRP (Fig. 5A; Table S1) (30), indicating that a slight intoxication results in global rather than specific changes.

Several studies indicate that DNA-damaging agents can disturb the redox state of a cell under certain conditions (31, 32). Redox is known to regulate a wide range of processes in the cell, including the binding ability of the Fur transcription factor(33). We therefore investigated if kin-delivery of CdiA-CT^o11^ affected the redox state of cells by repeating the monoculture setup with or without 5 mM Glutathione (GSH). GSH acts as a redox shunt and has been shown to protect against ciprofloxacin-induced redox damage (32). After 8 h, 6% of the cells in a *cdiBA-CT-I*^o11^ monoculture became YFP+ when GSH was absent (GSH−) as compared to 3% when GSH was present (GSH+) (Fig. 5B). After 10 h, the difference between GSH+/− was less pronounced (8% vs 6%), possibly due to GSH consumption (Fig. 5B). Populations with the *cdiBA*^STICK^ showed no increase in YFP+ positive cells in either condition (Fig. 5B). Thus, increasing the reducing capacity of the cell by the addition of GSH mitigates the cellular imbalance in redox state to some extent. Taken together, our results suggest that CdiA-CT^o11^ intoxication results in a shift in redox status, which in turn changes gene expression of the affected cells.

### Intoxication effects in kin and non-kin cells

A previous study identified a T6SS toxin with deaminase activity, which increased the mutation rate of intoxicated target cells (34). Theoretically, increasing the mutation rate in a small subpopulation of cells would facilitate adaptation to new niches (35, 36), without affecting the fitness of the entire population (37, 38). However, none of the genes involved in mutagenic repair (*dinB, umuDC*) were upregulated after 5 min of intoxication, even though 7- to 10-fold induction could be observed after 20 min (Fig. S7A). In addition, no increase in mutation rate could be observed in monoculture (Fig. S7B and C) or intoxicated cells with immunity that had survived intoxication (Fig. S7D and E), suggesting that kin delivery does not cause subpopulations of hypermutable cells.

DNA damage could also promote phage-mediated horizontal gene transfer. Prophages are induced by numerous stress signals, including the SOS response (39). In our data set, the strongest upregulation occurred in genes encoding prophage proteins. These prophages are unable to form functional phage particles, but overexpression of *ybcC* and *intD* can induce excision of the DLP12 prophage (40). To assess if intoxication induced DLP12 excision, we used quantitative PCR (qPCR) to detect phage excision as described previously (40). Expression of *cdiA-CT*^o11^ increased DLP12 excision ~40-fold, suggesting that CdiA-CT^o11^ intoxication indeed can result in prophage excision. However, we could not observe any prophage excision in monoculture (Fig. 5C), suggesting that either kin delivery does not promote prophage excision, or we are unable to detect such events due to the timing of excision/putative re-integration.

Taken together, our results suggest that delivery of a CdiA-CT^o11^ results in different outcomes among kin and non-kin cells. Although both effects are dependent on the toxic activity, the level of intoxication seems to guide the response to an altered redox state or to full induction of the DNA damage SOS response.

## DISCUSSION

Here, we show that kin-delivery of a CDI toxin with DNase activity generates population heterogeneity in terms of gene expression by intoxication of their siblings. This observation agrees with previous reports showing that toxin delivery among siblings, either through CDI or via the type 6 secretion system, can create heterogeneity in gene expression (6, 7). These previous reports indicated that toxin delivery among immune kin bacteria induces the RpoS-mediated stress response in some cells (6, 7). These studies did not untangle whether heterogeneity could arise from (i) the delivery event, (ii) the toxin-immunity complex acting as transcription factors, and/or (iii) the toxin activity. The distinction between these mechanisms is important, as, for example, in (i) all toxins delivered would have the same effect, whereas for (ii) and (iii) different toxins could exert different transcriptional changes. Here, we find that the observed heterogeneity is not caused by delivery *per se* (i), as no effect could be seen with the inactive toxin. Instead, our results suggest that the gene expression changes observed are specific to the toxin (iii) or toxin/immunity complex (ii) and that changes mediated by other toxins will likely be different. This is also supported by the finding that CDI toxins with other activities cannot induce the SulA reporter (Fig. S8A and B).

A remaining question is why only a subpopulation of cells is affected by the toxin. The data shown here suggest that the availability of non-occupied outer membrane receptor BamA proteins determines toxin import. This would suggest that under conditions where few outer membrane proteins are being produced, more CdiA toxins can be delivered. OmpC and OmpF are the most highly expressed outer membrane proteins in *E. coli*, forming pores that allow passive diffusion of hydrophilic molecules. The levels of these two porins are tightly regulated, and expression of OmpC is known to increase in response to acidic pH and high osmolarity (41, 42), conditions that vary, e.g., within the host. Thus, it is possible that kin-delivery mediated population heterogeneity occurs in specific conditions affected by the expression of these porins and other abundant outer membrane proteins.

Importantly, the changes inflicted in intoxicated cells by the DNase toxin differ between kin-cells and non-immune cells. Although *sulA* expression is induced in both, intoxication of kin-cells results in altered redox status, whereas non-immune cells experience DNA damage and induce the SOS DNA damage response. These findings are supported by the fact that kin delivery does not seem to increase mutation rates or promote horizontal gene transfer (Fig. S7C through E; Fig. 5C), whereas at least the latter is observed upon higher intoxication levels (resembling those experienced by non-kin cells). The lack of induction of classical SOS genes 5 min after intoxication and in monoculture, despite a clear induction of *sulA*, suggests that *sulA* is regulated by redox as well as LexA, explaining why the *sulA-sYFP2* reporter responds in monoculture.

What then could be the evolutionary purpose of heterogeneity in redox status in a population? One possibility is that redox heterogeneity could constitute a form of bet-hedging strategy. Previous findings suggest that CDI-mediated population heterogeneity is important for antibiotic tolerance, where some cells are able to survive antibiotic exposure due to growth arrest (7). In support of this, a recent study links cellular redox status to antibiotic susceptibility in *Pseudomonas* (43). However, we find that kin-delivery of CdiA-CT^o11^ does not increase antibiotic survival (Fig. S9). In addition, we did not see any increase in mutation rates in response to kin-intoxication. Thus, kin-delivery of toxins does not appear to be a general bet-hedging strategy. Nonetheless, it is, of course, possible that this particular toxin enables coping with other types of stress. More work is needed to shed light on the evolutionary role of kin-delivery of CDI-toxins.

Another possibility is that phenotypic heterogeneity via unequal toxin delivery is important for quasi-multicellular behavior. An important hallmark of this is to acquire heterogeneous responses to the same stimuli among genetically isogenic cells, i.e., polyphenism (44). Redox status is known to be important for multicellular behavior, regulation of virulence, and cellular responses to environmental cues (reviewed in reference 45). Thus, a heterogeneous redox status could allow for polyphenism in a bacterial population. Although the presence of polyphenism itself is not sufficient evidence for division of labor, we suggest that the presence of CDI toxins could form the basis of multicellular behavior, whether this is then used for division of labor or not.

A remaining question is how a DNase toxin changes the redox status of the targeted cell. As also antibiotics that target DNA, e.g., ciprofloxacin, have previously been shown to change bacterial redox status (31, 32), one might speculate that there is a common pathway through which DNA stress is sensed and forwarded to redox changes in bacteria. Future studies should be aimed at understanding how these processes are intertwined to get a better understanding of how bacteria respond to various intoxications.

## MATERIALS AND METHODS

### Bacteria and growth conditions

All bacteria used in this study are derivatives of *E. coli* MG1655 and are listed in Table S2. All plasmids used in the study are listed in Table S3. Assays were performed in M9 minimal media with 1% glycerol and 0.2% cas-amino acids (M9-gly-CAA) from Gibco. Overnight cultures prior to assays were grown in Luria broth (LB) (1% NaCl, 1% Tryptone, 0.5% yeast extract, Gibco). L-Arabinose was added to a 0.2% final concentration unless stated otherwise. Liquid cultures were grown at 37°C with 200 rpm shaking. Unless otherwise specified, agar plates were made from LB with 1.5% agar. Plates were incubated overnight at 37°C. Antibiotics were used as follows: 50 mg/L Kanamycin (KAN), 12.5 mg/L Chloramphenicol (CAM), and 100 mg/L Ampicillin (AMP).

### Competition assay

Inhibitor and target strains were grown independently, without antibiotics, to OD_600_ ~0.7. The cultures were mixed 5:1 (inhibitor:target cells) and grown at 37°C, shaking. Samples were diluted serially and spotted on LB (Fig. S1) or on M9-gly-CAA + L-arabinose (Fig. S3B, S4B, S6) plates with or without KAN for colony counts at every time point. For recovery (as in figures [Fig. 3 and 4; Fig. S6]), samples were diluted 1:2 in fresh media containing arabinose for 30 min prior to plating and flow cytometry. Flow cytometry was performed using a BFP-dependent trigger to distinguish target (BFP+) cells from inhibitor (BFP−) cells unless otherwise indicated. Competitive index (CI) was calculated as the change in ratio between inhibitor and target cells over time: CI = (cfu_inhibitor tX_/cfu_target tX_)/(cfu_inhibitor t0_/cfu_target t0_). Statistical significance was determined using Student’s *t*-test.

### Monoculture experiment

Overnight cultures were diluted 1:1,000 into 10 mL fresh medium. Cultures were grown for 11 h with sampling for OD_600_ and flow cytometry measurements at time points −1, 0, 2, 4, 6, 8, 9, 10, and 11 h post-dilution, where −1 h simply indicates pre-dilution. To account for varying baselines in YFP fluorescence, YFP thresholds were set relative to 1% YFP+ cells in the corresponding *cdiBA*^STICK^ culture for each time point, unless otherwise specified. Statistical significance was determined using two-way ANOVA with Tukey’s post hoc test.

### Immunity dilution assay

Strains were grown for approximately 15 h before dilution to OD_600_ ~0.003 and split into two tubes. Target cells were grown to an OD_600_ ~0.5, then L-arabinose was added to one of the tubes. Inhibitor cells were grown for the same time, but with no L-arabinose added. Fifteen minutes after L-arabinose addition, all cultures were spun down and resuspended in an equal volume of fresh media. This constituted cycle 0. The culture was diluted 1:5.3 in 20 mL of fresh media, and the resulting culture was re-grown for 2 h to an OD_600_ ~0.7. This constituted cycle 1. The same procedure was repeated for a total of eight cycles with sampling at each cycle for (i) OD_600_ measurement (1 mL), (ii) a 1:1 competition with the inhibitor cells for 1 h (3 mL), and (iii) western blot (2 mL). The competitive indexes were determined as described above. At cycle 8, the sample was diluted in twice the volume as used for the other cycles (40 mL), and the dilution was split into two flasks. The first flask was treated as in all other cycles. The second flask was grown to OD_600_ ~0.5, when L-arabinose was added to the culture for 15 min before being subjected to the same tests: (i)–(iii) as the other samples.

### Transcriptomics—sample preparation

Strains with *lacA::cdiBA-CT-I*^o11^ and either an arabinose-inducible pBAD33::*cdiA-CT*^o11^ or a pBAD33^empty^ (EVC) were grown to OD_600_ ~0.5 in M9-gly-CAA supplemented with CAM (*n* = 3 biological replicates). The cells were centrifuged at 3,000 × *g* for 10 min and resuspended in new media before samples (t0) were taken as described below. Arabinose was added, and the cultures were incubated for 20 min (t20). At each time point, a 350 µL sample was mixed with 700 µL RNAprotect (Qiagen), and 200 µL was used for viable counts. Viable counts were spotted on M9-gly-CAA plates supplemented with 1% glucose and CAM. Samples in RNAprotect were incubated for 40 min, shaking at room temperature. RNA was then isolated using the RNeasy kit from Qiagen according to the manufacturer’s instructions (Qiagen). RNA quality and quantity were determined using NanoDrop and Qubit. RNA was sent to SciLifeLab, Stockholm, for library preparation and sequencing. The quantity and quality of isolated RNA were determined using a Fragment Analyzer with a DNF-471 Standard Sensitivity RNA kit (Agilent Technologies). All samples had an RQN value of >9, except for one sample with an RQN of 7.1. Sequencing libraries were prepared from 100 ng total RNA using the Illumina Stranded Total RNA library preparation kit with Ribo-Zero Plus treatment (cat# 20040525/20040529, Illumina Inc.). Unique dual indexes (cat# 20040553/20040554, Illumina Inc.) were used. The library preparation was performed according to the manufacturer’s protocol (# 1000000124514). The quality of the generated sequencing libraries was evaluated using a Fragment Analyzer with a DNF-910 dsDNA kit (Agilent Technologies), and quantified by qPCR using the KAPA Library Quantification Kit for Illumina (Roche) on a CFX384 Touch instrument (Bio-Rad) prior to cluster generation and sequencing. Sequencing was carried out on an Illumina NovaSeq X Plus system using a 10B flow cell and XLEAP-SBS sequencing chemistry, with paired-end 150 bp read length.

### Transcriptomics—data analysis

Raw reads were mapped with minimap2 (46) to SK1371 (*E. coli* MG1655/pBAD33), annotated with prokka (47). The number of reads mapping to each gene was generated using featureCounts (48). Differential expression and principal component analyses were performed with DESeq2 (49). All comparisons were performed against the empty vector control for each time point. Genes with a *P*-adj value <0.01 were considered to be differentially expressed (Table S1).

## Data Availability

All raw data for the manuscript are available at https://figshare.com/s/7c41161daa798c92b48a.

## SUPPLEMENTAL MATERIAL

Supplemental file mbio.02083-25-s0001.pdf Supplemental text, figures, and tables.

Table S1 mbio.02083-25-s0002.xlsx Transcriptomics data.

ASM does not own the copyrights to Supplemental Material that may be linked to, or accessed through, an article. The authors have granted ASM a non-exclusive, world-wide license to publish the Supplemental Material files. Please contact the corresponding author directly for reuse.
