# Supplementary material for: A delivered DNase toxin creates population heterogeneity through transient intoxication of siblings
Source: mBio. 2025 Sep 22;16(11):e02083-25. doi: 10.1128/mbio.02083-25 (PMC12607911; doi:10.1128/mbio.02083-25)
Supplement: Supplemental file — Supplemental text, figures, and tables. [file mbio.02083-25-s0001.pdf]

Supplementary material for: A delivered DNase toxin creates population heterogeneity through transient intoxication of siblings.

Hanna Eriksson, Susan Schlegel and Sanna Koskiniemi*

This file contains:

Supplementary methods

Supplementary figures 1-9

Supplementary tables 1-4

Supplementary references

Supplementary methods

Strain constructions

Oligos used for strain constructions are listed in Table S4.

Chromosomal constructs

Strains encoding chromosomal *cdiBA-CT-I* ^{o11} were generated by amplifying the *cdiA-CT-I*^{o11} from pDAL930 [1] using oligos SK2071 and SK2077. This PCR product was fused to a KAN resistance marker (amplified from pKD4 using oligos SK2078 and SK2074), using overlap-extension PCR with oligos SK2071/2074. The PCR product was recombined into the *cdiBA-CT-I* ^{STICK} construct using lambda red recombination [2]. Colonies with the correct antibiotic resistance were screened with PCR using oligos 2100 and 2076 and colonies with the correct insert were sent for sequencing for verification using sequencing primer SK2100.

Strains with *sulA-sYFP2* reporter carrying the KAN resistance marker was made by replacing the *cat* gene with the *kan* gene from pKD4. The gene encoding *kan* was amplified using oligos SK1082 and SK1083 on pKD4 and inserted into SK807 using lambda red recombination.

Chromosomal constructs were moved between strains by P1 transduction [3]. Unwanted antibiotic cassettes were removed by pCP20-induced FLP expression as described previously [2].

Plasmid constructs

Plasmids encoding *bamA* and *yciB* were created by amplifying *yciB* from the MG1655 chromosome using oligos SK2225 and SK2226. PCR products of the correct size were cloned

into the pZS21::*bamA* plasmid (from [4]) using *Xba*I and *Sal*I. Successful transformants were verified using oligos SK2231-2232.

To create pSK5111 ((pBAD33 *cdiI*^{o11}-*mTagBFP2* (5'UTR: AGAGAAATACTAG$^{(-1)}$)) *cdiI*^{o11} was amplified from pDAL930 using oligos SK1785 and SK2783. *mTagBFP2* was amplified using oligos SK1778 and SK1779. *mTagBFP2* was fused transcriptionally to *cdiI*^{o11} using the *Bgl*II restriction site and the resulting fragment (*cdiI*^{o11}-*mTagBFP2*) was cloned into pBAD33 using *Eco*RI and *Hind*III.

To create pSK5115 (pBAD33 *cdiI*^{o11}-α-*mTagBFP2*, (5'UTR: AAAGAAATACTAG$^{(-1)}$)), *cdiI*^{o11} was amplified from pDAL930 using oligos SK2781 and SK2782, introducing a C-terminal ALFA-tag (-α) and a suboptimal 5'UTR. *mTagBFP2* was amplified using oligos SK1778 and SK1779. *mTagBFP2* was ligated to *cdiI*^{o11}-α using the *Bgl*II restriction site and the resulting fragment (*cdiI*^{o11}-α-*mTagBFP2*) was cloned into pBAD33 using *Eco*RI and *Hind*III.

To create the pBAD33-variants pSK5244 (medium) or pSK5248 (weak), pBAD33 was amplified using oligos SK2777 and SK2796 or SK2778 (outbound PCR) respectively. The resulting PCR product was digested with *Kpn*I and ligated with T4-DNA ligase.

To create pBAD33^{weak}::*cdiI*^{o11}, the insert was amplified using oligos SK2593 and SK2594 from SK6861, followed by digestion with *Kpn*I and *Hind*III and ligation into pBAD33(weak). To create pBAD33::*cdiA-CT*$^{o11(D198A)}$ with or without an -α tag, *cdiA-CT*$^{o11(D198A)}$ was amplified from pSK4896 using Sk2599/2601 or SK2599/2600 respectively. For the latter, a second round of PCR was performed using oligos SK2599 and SK2787 to introduce a *Hind*III

site into the PCR product. The PCR products were cloned into pBAD33(medium) using *Kpn*I and *Hind*III.

pDAL930::*cdiBA*^{EC93}-*CT*^{D198A}-*I*^{o11} (containing the inactive CdiA-CT$^{o11(D198A)}$ mutation described in [1], here denoted as o11*) and pDAL930- *cdiBA*^{EC93}-*CT*^{o11}-α-*I*^{o11}-*HA* were created using lambda red recombination and *pheS* counter selection essentially as described in [1]. Briefly, *CT*^{o11}/*cdiI*^{o11} was amplified from pSK478 using oligos SK2784 + SK2794 (fragment 1) and SK2793 + SK690 (fragment 2), introducing the inactivating mutation. Fragments were combined using overlap extension PCR and oligos SK2784 and SK690 and recombined into pCH10163. Successful recombinants were selected on agar plates containing 4-Chloro-D-phenylalanine and CAM. pDAL930- *cdiBA*^{EC93}-*CT*^{o11}-α-*I*^{o11}-*HA* was created in a 2-step process: First, the C-terminal α-tag was introduced using oligos SK2784 + SK2786 (fragment 1) and SK2785 + SK690 (fragment 2); fragments were combined using overlap extension PCR and oligos SK2784 and SK690. In a second step, this construct was used to introduce an HA-tag on the immunity gene using essentially the same procedure and oligos SK2784 + SK2792 (fragment 1) and 2791+ SK690 (fragment 2). Comment: The HA-tag was not used in the end.
Plasmids were extracted from construction strains by plasmid purification using the GeneJET Plasmid Miniprep Kit (ThermoFisher Scientific, US) and transformed into clean strain backgrounds by electroporation.

SDS-PAGE/Western blot

Cells were harvested, dissolved in SDS loading dye (50 mM Tris pH 6.8, 1% SDS (w/v), 1% Triton X-100, 10% glycerol, 0.2% bromophenol blue) and incubated for 10 minutes at 75°C. DTT was added to a final concentration of 125 mM and chromosomal DNA was digested by benzonase treatment for 10 min at room temperature. Remaining debris was pelleted by

centrifugation at 16000 x g for 5 min at room temperature. For visualization of undelivered, full-length CdiA-CT^{o11}-α, proteins were separated using NuPAGE 3-8% Tris-Acetate gel with Tris-acetate running buffer and HiMark pre-stained protein standard (Invitrogen) as size marker. For visualization of CdiI^{o11}, CdiI^{o11}-α, and CdiA-CT^{o11*}-α, proteins were separated using NuPAGE 12% Bis-Tris gels or 4-12% Bis-Tris gels with MES running buffer and PageRuler^{TM} plus prestained protein marker (Thermo fisher) as size marker. Proteins were transferred to a PVDF-membrane using iBlot^{TM} pvdf transfer stacks (Invitrogen). CdiI^{o11} was detected using a custom-made rabbit anti-o11-CdiI antibody, followed by IRDye 800CW Goat anti-rabbit secondary antibody (licor). CdiI^{o11}-α was detected using the Fluo-tag X2 anti-ALFA, Alexa Fluor 647 nanobody (Nanotag). To enhance detection, CdiA-CT^{o11*}-α was detected using recombinant mouse anti-ALFA antibody (Nanotag) followed by IRDye 680 Goat anti-mouse secondary antibody (licor). RNA polymerase beta (RpoB) was detected using Rb mAB to RNA polymerase beta (RpoB) (abcam) followed by IRDye 800CW Goat anti-rabbit secondary antibody (licor). All incubations were done in blocking buffer (5% (w/v) milk powder in PBS) according to standard procedure. Proteins were visualized using the Licor Odyssey imaging system (licor).

Protein stability

To estimate the stability of CdiI^{o11} and CdiA-CT^{o11}-α, cultures were grown in M9-gly-CAA to late exponential phase and SHX was added to a final concentration of 1 g/L. Samples were taken at the indicated time points (0 = before SHX-addition). Cells were harvested, resuspended in 4x LDS loading dye supplemented with 125 mM DTT and separated on NuPAGE 4-12% BisTris gels with MES running buffer. The proteins were detected using western blot as described above.

Flow cytometry

Samples were diluted in sterile filtered PBS and measured in a MACS flow cytometer (Miltenyi, Sweden) at a flow rate of 1000-2000 events/s. For each sample, 100,000 events were measured wherever possible. Depending on the sample composition, either a side scatter- or a BFP- or RFP- trigger was used. YFP, BFP and RFP were excited with a blue laser (488 nm; bandpass filter 525 nm channel B1) a violet laser (405 nm; bandpass filter 450 nm channel V1) and a yellow laser (561 nm, bandpass filter 620 nm channel Y2) respectively using MACSQuant VYB (Miltenyi Biotec). Data was analysed using FlowJo™ software version 10.9.0 from Becton Dickinson & Company (BD) 2006-2023.

Microscopy

Samples were spotted on an 1 % low-melting agarose pad and imaged using the 100 x objective of a Ti Microscope fluorescence microscope with an Andor Zyla camera. For microscopy timelapse, an agarose pad containing M9-gly-CAA, 0.2% L-arabinose and 50 µg/ml kanamycin and the following settings were used: phase images, 200 ms exposure with a 12.0 V Nikon Ti "Mono" filter. BFP images, 10 ms exposure with a 3.0 V Nikon Ti DAPI filter. YFP images, 40 ms exposure with a 3.0 V Nikon Ti FITC filter. Microscopy images were analyzed using FIJI (Fiji is just ImageJ) version 2.0.0-rc-69/1.52p

Whole genome sequencing

Genomic DNA was prepared from 20 isolated colonies using Genomic-tip 100/G columns (Qiagen). PCR-free library construction and whole-genome re-sequencing was performed by the BGI sequencing facility (Hong Kong, China) using the HiSeq 4000 PE150 sequencing system (Illumina Inc., San Diego, CA, USA). Illumina analysis pipeline was used for image analysis, base calling and quality score calibration. Raw sequence reads were filtered and

exported as FASTQ files. Sequence reads were mapped onto the *E. coli* MG1655 genome using the CLC Genomics workbench (Qiagen), and SNP/DIP/structural variant detection analyses were used to identify unique mutations in the isolates.

Quantitative PCR

Quantitative PCR (qPCR) was performed using SYBRgreen qPCR master mix according to the instructions of the manufacturer (Thermo Scientific). To quantify the relative abundance of integrated phage DLP12 in the *E. coli* MG1655 chromosome, cells were grown as for the transcriptomics experiment above, but instead of harvesting RNA, genomic DNA was isolated using the GeneJet Genomic DNA purification kit (Thermo Scientific). Relative abundance of DLP12 integration site and the *purA* ORF was determined using oligos SK3034-3035 and SK3036-3037 respectively, and the ΔΔCt method.

Persister assay

Overnight cultures were diluted 1:1000 into 10 ml fresh M9-gly-CAA medium. Cultures were grown for 10 h with sampling for OD_{600} and flow cytometry measurements at time points 0, 4, 6, 8 and 10 h post-dilution. At 6 h and 8 h, 3 ml of each sample was transferred to a 50 ml Falcon tube. A small aliquot of each sample was used for viable counts (t0) before 50 mg/L KAN was added to the remaining sample. Samples were incubated at 37 °C, shaking and plated for viable counts after 3 and 5 h. Statistical significance was determined through Student’s t-test.

Supplementary figures

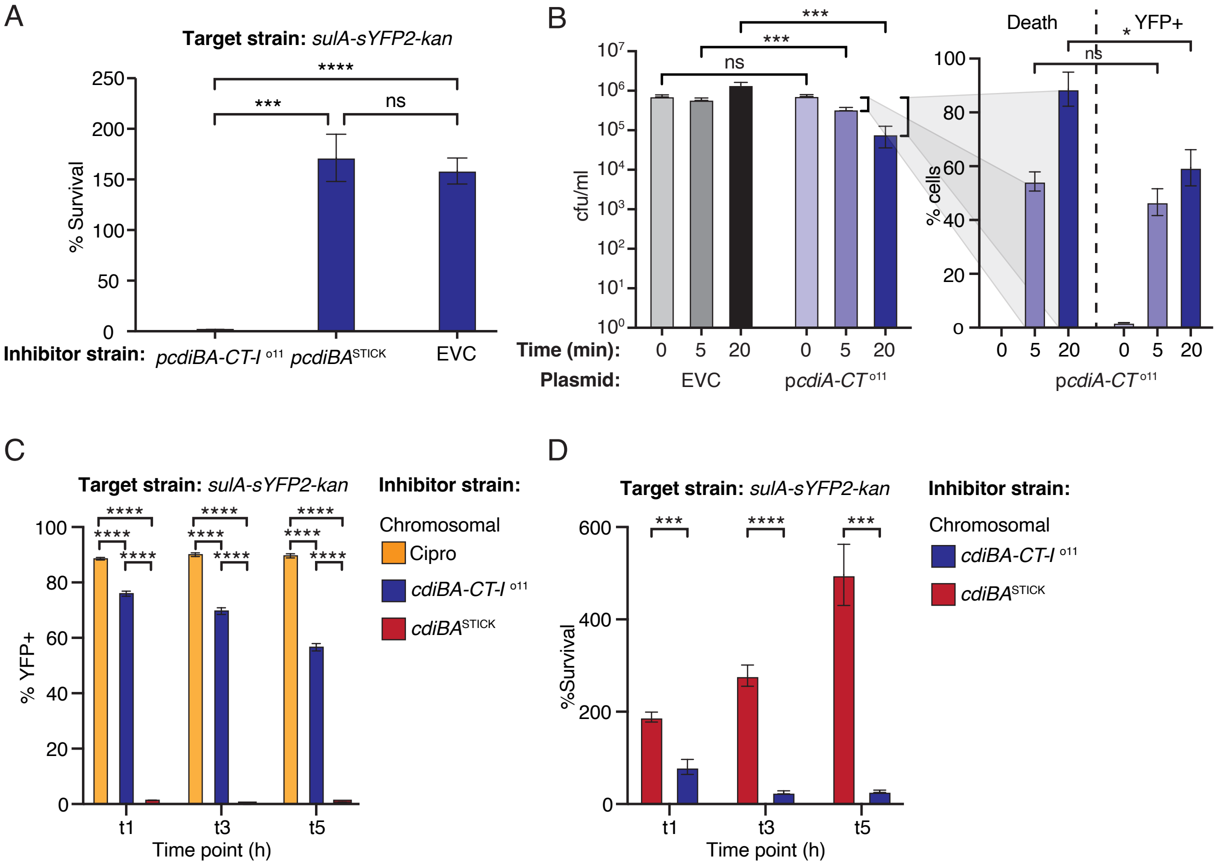

Figure S1. DNase toxins inhibit growth of unprotected MG1655 cells. A) MG1655 cells with p*cdiBA-CT-I* ^{o11}, p*cdiBA*^{STICK} or empty vector grown in co-culture with MG1655 *lacA-kan* target cells at 5:1 ratio for 1 h in M9-gly-CAA. % Survival was calculated as the change in cfu/mL of target cells over time. N = 6 biological replicates. **B)** Cfu/mL of MG1655 cells with pBAD33 (EVC) or pBAD33::*cdiA-CT* ^{o11} (p*cdiA-CT* ^{o11}) grown in the presence of 0.2% L-arabinose for 0, 5 or 20 min (right). Cell death was assessed as the reduction in colony forming units and the percentage of dead cells was calculated as as the loss in viability during induction, relative to the starting cfu/ml. (%Death = (cfu/mL$_{t0}$ - cfu/mL$_{tX}$)/cfu/mL $_{t0}$. Illustration of how % death was calculated from this data (right) and %YFP+ cells in the p*cdiA-CT* ^{o11} population (left). N= 6 biological replicates. **C-D)** MG1655 cells with *cdiBA-CT-I* ^{o11} or *cdiBA*^{STICK} inserted downstream of *lacA* on the chromosome grown in co-culture with MG1655 *sulA-sYFP2-kan* target cells at 5:1 ratio for 5 h in M9-gly-CAA. N = 6 biological replicates. Percentage of YFP+ cells (C) or survival (D) was determined through viable counts or flow cytometry respectively. Error-bars are SEM. Statistical significance was determined through Student's t-test for (A-D).

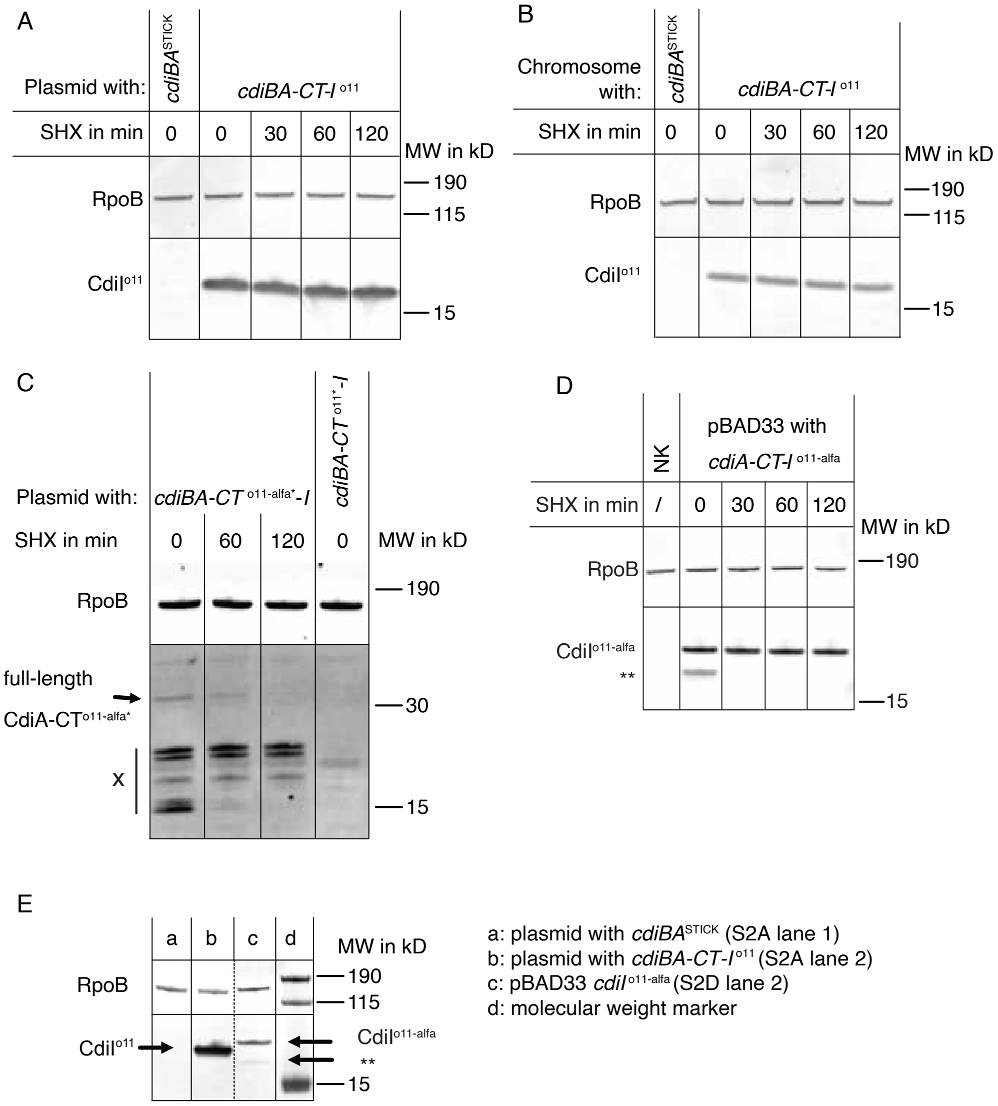

Figure S2. Stability of CdiA-CT^{o11} and CdiI^{o11}. A-B) Stability of CdiI^{o11} determined through western blot using anti-CdiI^{o11} antibodies after SHX addition. CdiI^{o11} is expressed from its native promoter on a medium copy *pWEB::cdiBA-CT-I* ^{o11} plasmid (A) or from the chromosome (B). **C)** Stability of an inactive D198A (*) variant [1] of CdiA-CT^{o11}-α determined through western blot using an anti-α antibody after SHX addition. CdiA-CT^{o11}-α is expressed from pBAD33 under an arabinose inducible promoter. x indicates putative proteolytic degradation or internal expression products of CdiA-CT^{o11}-α. Full-length CdiA-CT^{o11} is indicated by an arrow. **D)** Stability of CdiI^{o11}-α determined through western blot using anti-α nanobodies after SHX addition. CdiI^{o11}-α is expressed from an L-arabinose inducible promoter on pBAD33 with a weak SD sequence. ** indicates internal expression products of CdiI^{o11} which can only be seen at low level of expression as can be seen in Fig. S2E. **E)** Comparative western blot of CdiI^{o11} and CdiI^{o11}-α. CdiI^{o11} is expressed from its native promoter on a medium copy plasmid as in Fig. S2A (pWEB::*cdiBA-CT-I* ^{o11}), CdiI^{o11}-α from pBAD33::*cdiI* ^{o11} as in Fig. S2D. Both immunities were detected with an anti-CdiI^{o11} antibody. Full-length CdiI^{o11} with or without an α tag is indicated. ** indicates an internal expression product of CdiI^{o11} only visible at low levels of CdiI^{o11} expression. **A-E)**: Levels of *E. coli* RNA polymerase beta (RpoB) were assessed as loading control.

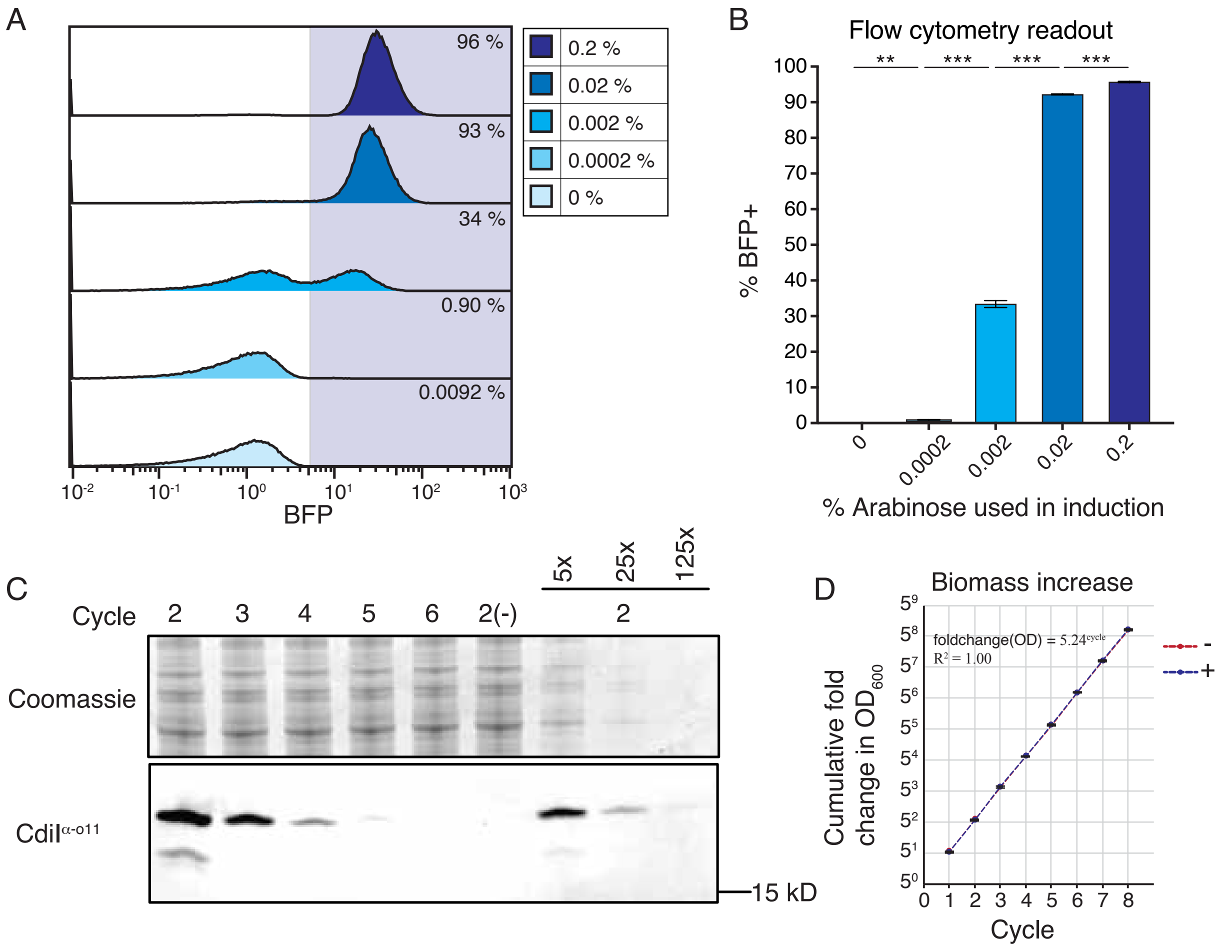

Figure S3. CdiI levels upon induction of immunity. A-B) Expression of *mTagBFP2* from an arabinose inducible pBAD promoter at different concentrations of L-arabinose determined through flow cytometry. Left: Flow cytometry histogram of pBAD-*cdiI* ^{o11}-α -*mTagBFP* cells after 1 h of induction with 0-0.2% L-arabinose. The blue box shows the BFP+ gating used. Right: Quantification of BFP+ cells shown in the histogram (in %). (N = 3 biological replicates). Error bars show SEM, statistical significance was assessed using Student's T-test, **<0.01, ***<0.001. **C)** Western blot showing CdiI^{o11}-α levels during immunity dilution experiment in Figure 2C but with diluted samples to improve visualization of immunity dilution at early timepoints (bottom). CdiI^{o11}-α was detected using anti-α nanobodies. To estimate reduction in CdiI^{o11}-α from cycle to cycle, the sample from cycle 2 was diluted 5x, 25x and 125x and analyzed on the same gel. Before western blotting, the SDS gel was split and the top part was Coomassie-stained to serve as loading control (top). 2(-) indicates cells from cycle 2, sampled from cultures where arabinose was not added. **D)** Biomass increase over the cycles estimated as cumulative fold increase in OD_{600} values from the beginning of cycle 1 to the end of the cycle indicated. (N = 3 biological replicates), error bars show SEM.

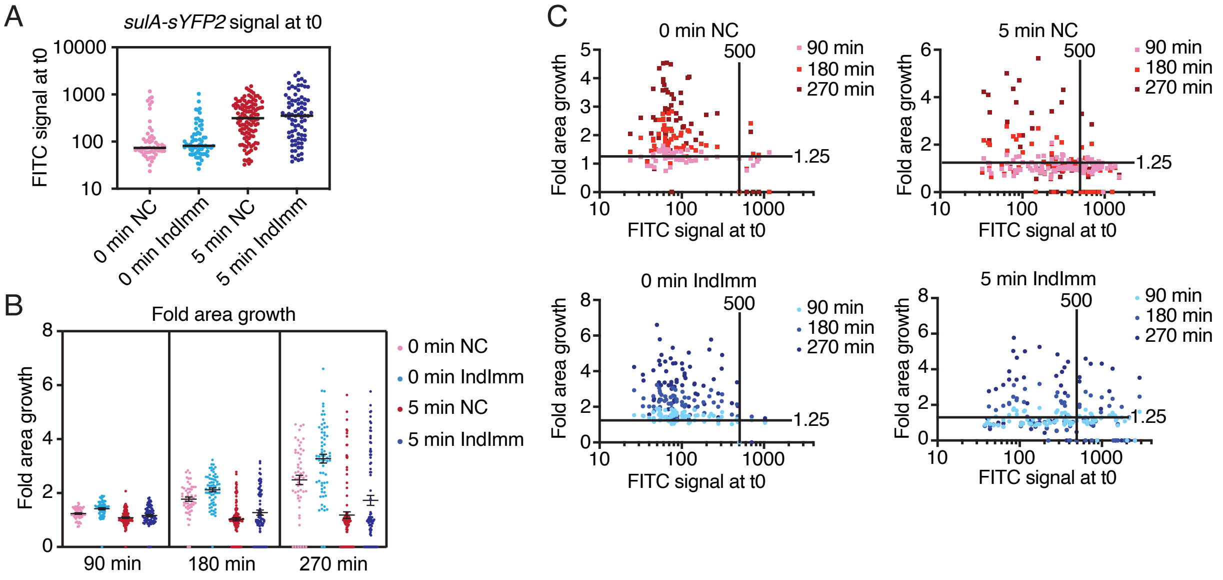

Figure S4. Time-lapse microscopy of individual target cells upon mixing with inhibitor cells. A) YFP (FITC) levels and **B)** Fold area growth of individual target cells with inducible p*cdiI* ^{o11} immunity (IndImm) or empty vector (NC) after 0 or 5 minutes interaction with *cdiBA-CT-I* ^{o11} inhibitor cells immediately after (A) or 90, 180 or 270 minutes after absorption into agarose pad containing arabinose. **C)** Fold area growth at t90, t180 and t270 over YFP (FITC) signal at t0 for each sample. Lines indicate thresholds used for quantification shown in figure 3C. Fold area growth refers to the total area of daughter cells at the later time point, divided by the area of the mother cell. N = 53, 66, 109 and 82 cells for 0 min NC, 0 min IndImm, 5 min NC and 5 min IndImm, respectively.

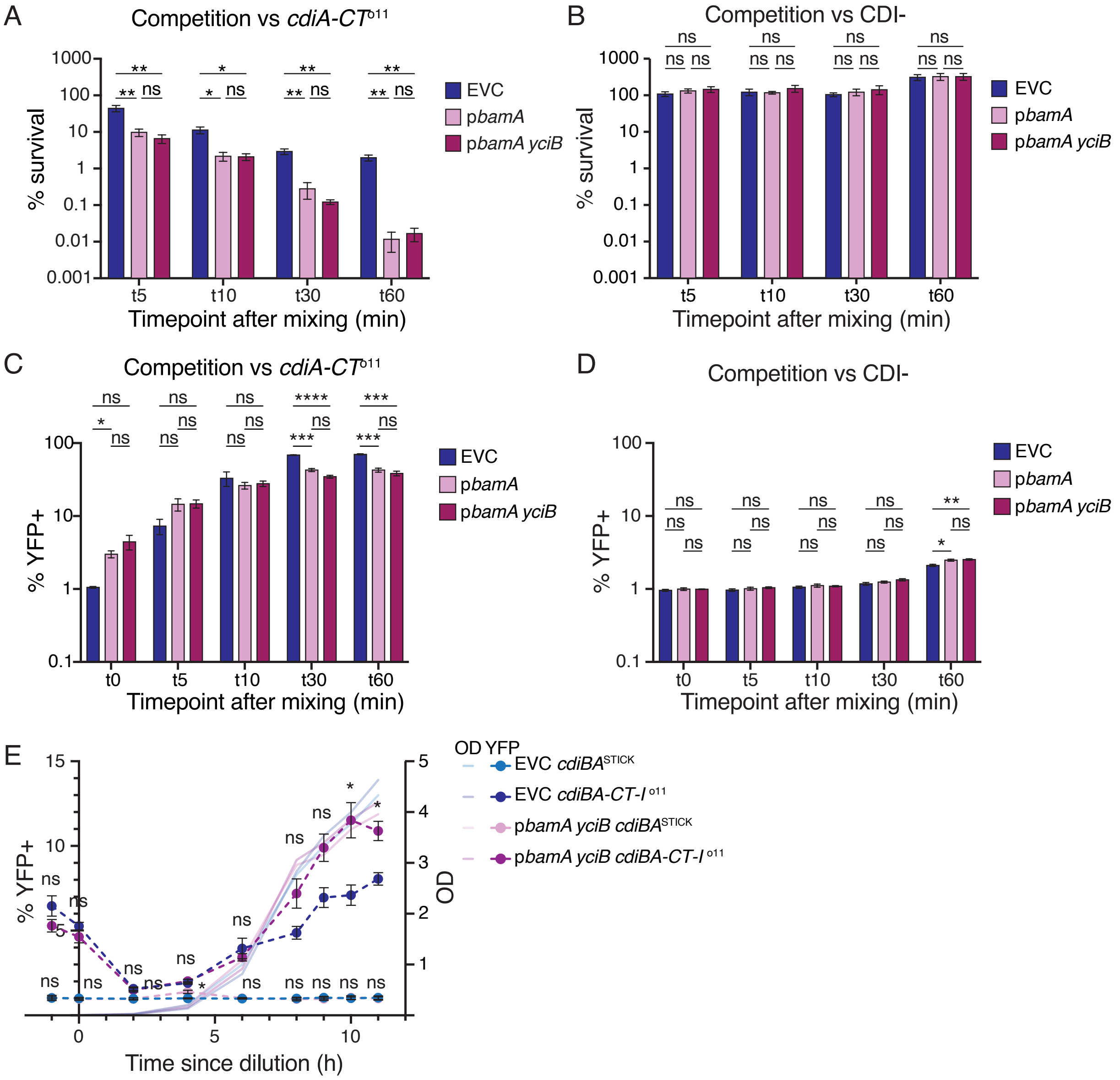

Figure S5. SOS induction in target cells upon mixing with p*cdiBA-CT-I*^{o11} inhibitor cells. A-B) Percentage of surviving target cells with an empty vector (EVC, blue), p*bamA* (purple), or p*bamA-yciB* (red) after 5, 10, 30, or 60 min of co-culture with MG1655 inhibitor cells with (A) or without (B) p*cdiBA-CT-I*^{o11}. **C-D)** Percentage of YFP+ MG1655 *sulA-sYFP2* p*bamA* or p*bamA-yciB* target cells in the experiments in A-B. **E)** Percentage of YFP+ cells in monocultures of MG1655 cells with chromosomal *cdiBA-CT-I*^{o11}(dark colors) or p*cdiBA*^{STICK} (light colors) supplemented with p*bamA-yciB* (purple) or empty vector (blue). Average OD_{600} values for the cultures are shown as shaded lines. Error-bars are SEM. Statistical significance was determined through A-D) Student's t-test, and E) two-way ANOVA with Tukey's posthoc test. * <0.05, ** <0.01, *** <0.001, ****<0.0001. N= 6 biological replicates for A-D and 6, 10 (EVC strains, p*bamA-yciB* strains, respectively) for E.

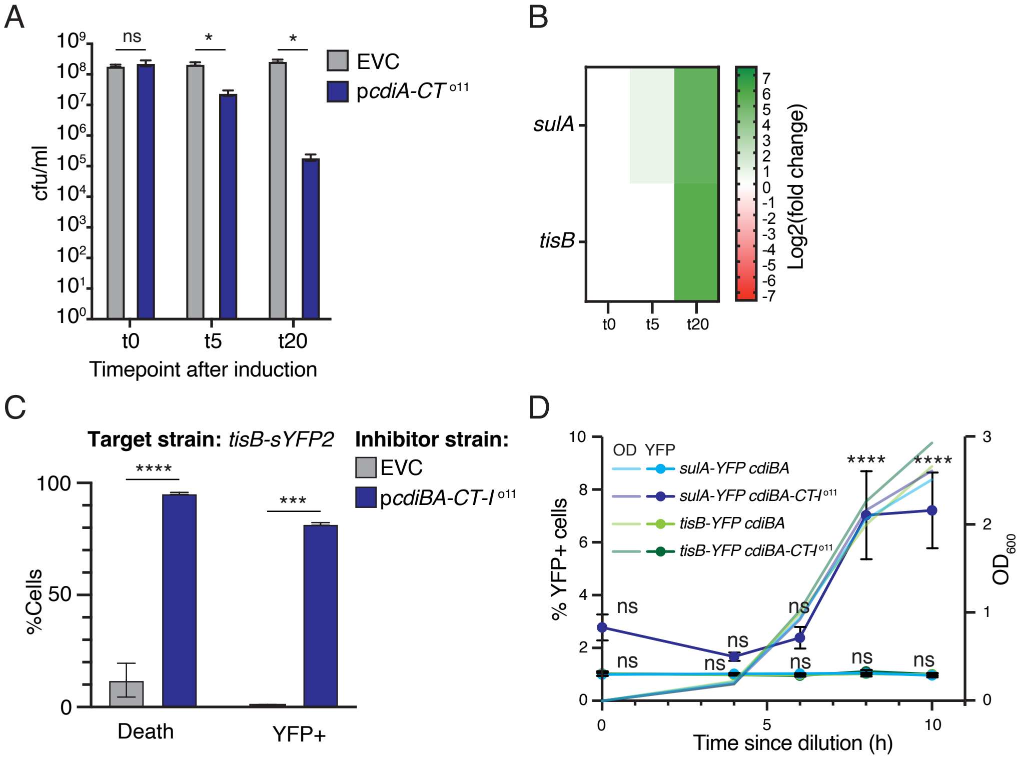

Figure S6. Transcriptomics and *tisB-sYFP2* reporter. A) Viability of *E. coli* cells 5 and 20 min after CdiA-CT^{o11} induction by L-arabinose in the samples analyzed with transcriptomics (Fig. 5A). *E. coli* MG1655 cells with pBAD33::*cdiA-CT*^{o11} (p*cdiA-CT*^{o11}) or pBAD33^{empty} (EVC) were induced with 0.2% L-arabinose. Viability was monitored as CFU/ml using serial dilution and plating. (N = 3 biological replicates). **B)** Heatmap showing relative mRNA levels of *sulA* and *tisB* mRNA 5 and 20 min after CdiA-CT^{o11} induction. **C)** % dead and % YFP+ target cells after co-culture with p*cdiBA-CT-I*^{o11} (blue) or EVC (grey) inhibitor cells for 60 minutes M9-gly-CAA. Error bars are SEM. (N = 6 biological replicates). **D)** Time-resolved enumeration of growth and YFP+ cells in monocultures grown in M9-gly-CAA for 10 h. *E. coli* MG1655 with the *tisB-sYFP2* (green) or the *sulA*-sYFP2 (blue) reporter and *cdiBA-CT-I*^{o11} (dark colors) or *cdiBA*^{STICK} (light colors) were used. (N = 6 biological replicates). Error bars are SEM. Statistical significance was determined through A and C) Student's t-test. D) Significance is relative to the respective *cdiBA*^{STICK} strain, determined through two-way ANOVA with Tukey's posthoc test. * <0.05, ** <0.01, *** <0.001, ****<0.0001.

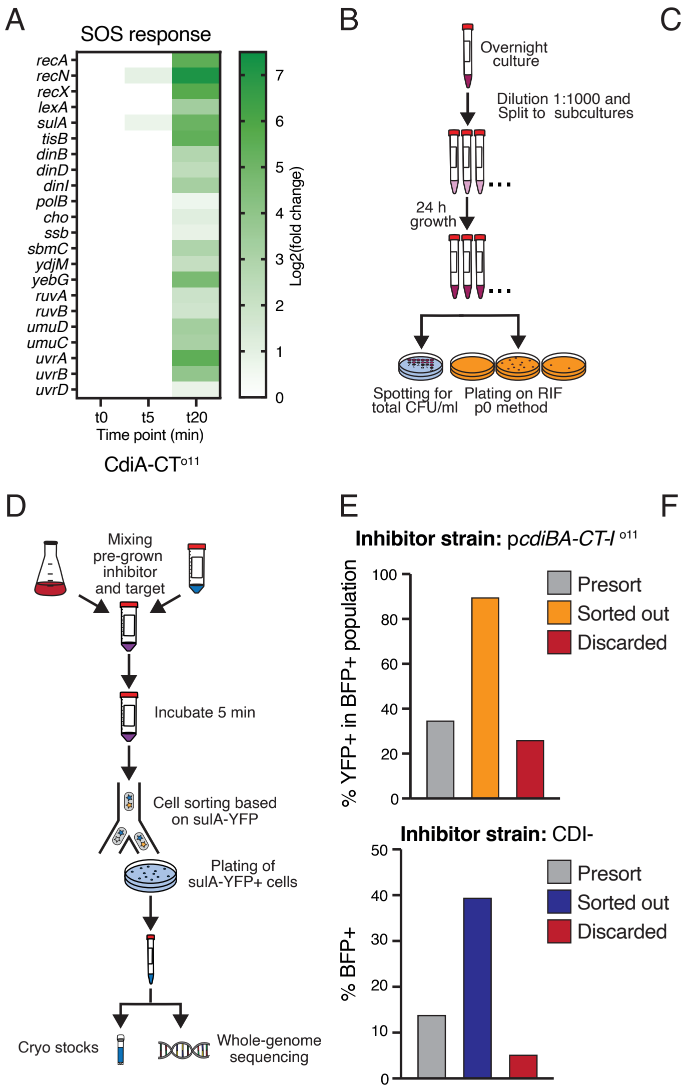

F

Frequency of SNPs		
Closest gene	CDI-	pcdiBA-CT-I o11
ycbF	0.1	0
sspA	0	0.1
gadX	0.1	0
**Total number of SNPs observed**	**2**	**1**

Frequency of Structural Variants		
Closest gene	CDI-	pcdiBA-CT-I o11
ykgN	0.6	0.4
ybhB-ybhC (intergenic)	0.6	0.5
ybhB	0.4	0.5
stfE-ycfK (spans several genes)	0.3	0.3
fadK	0.7	0.3
insB-yrhA (intergenic)	0.7	0.4
rrfB-murB (intergenic)	0.1	0
**Total number of Structural Variants observed**	**34**	**24**

Figure S7. CdiA-CT^{o11} intoxication does not increase the mutation rate. **A)** Heat-map showing relative mRNA levels for genes involved in DNA repair and mutation accumulation. **B)** Overview of fluctuation test performed in C. **C)** Relative mutation rate between *cdiBA-CT-I* ^{o11} and CDI- strains measured through a classical fluctuation test plated on M9-gly-CAA plates supplemented with 100 mg/L rifampicin. (N = 4 biological replicates). Error bars are SEM. Statistical significance was determined through Student's t-test. **D)** Overview of sorting of intoxicated cells for whole-genome sequencing in E and F. **E)** Efficacy of sorting *sulA*-YFP+, BFP+ cells from a 5 min inducible immunity competition against p*cdiBA-CT-I* ^{o11} (top) or CDI- (bottom) as described in Fig 3AB. Presort, Sorted out and Discard denote samples taken before sorting, and from the positive and the negative compartments of the sorting cartridge, respectively. Gating for sorting was set to YFP+ within BFP+ (top) and BFP+ (bottom) respectively. **F)** Mutations (SNP or structural variants) detected in 10 colonies isolated after the sorting in D, E.

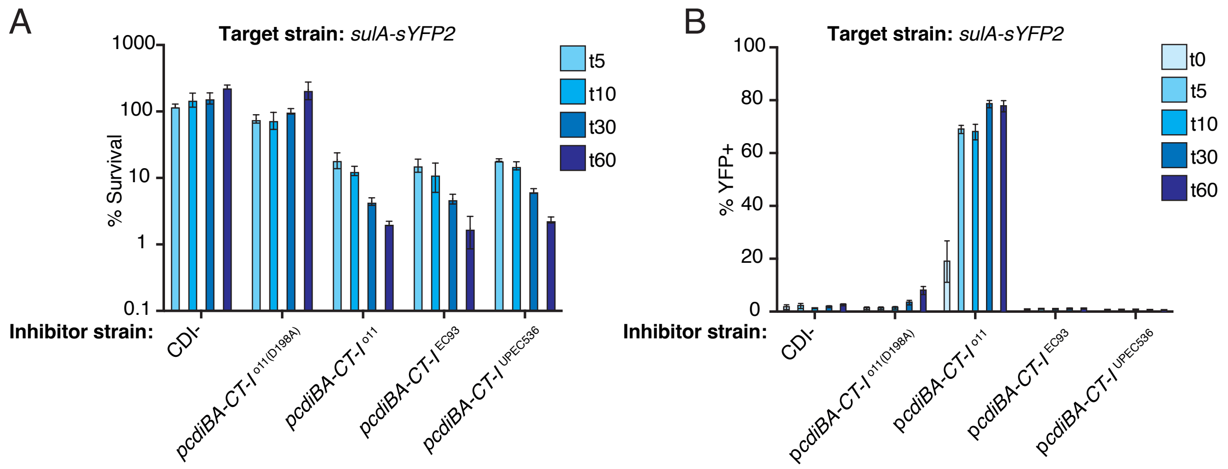

Figure S8. Induction of the *sulA-sYFP2* reporter is specific for the CdiA-CT^{o11} toxic activity. A-B) MG1655 cells with no CDI, p*cdiBA-CT-I* ^{o11}, p*cdiBA-CT-I* $^{o11(D198A)}$ (inactive variant), p*cdiBA-CT-I* ^{EC93} or p*cdiBA-CT-I* ^{UPEC536} (inhibitor) were grown in co-culture with MG1655 *lacA-kan*, *sulA*-sYFP2 target cells at 5:1 ratio for 1 h in M9-gly-CAA. Percentage of survival % (A) or YFP+ cells (B) was determined through viable counts or flow cytometry. (N = 3 biological replicates). Error bars are SEM.

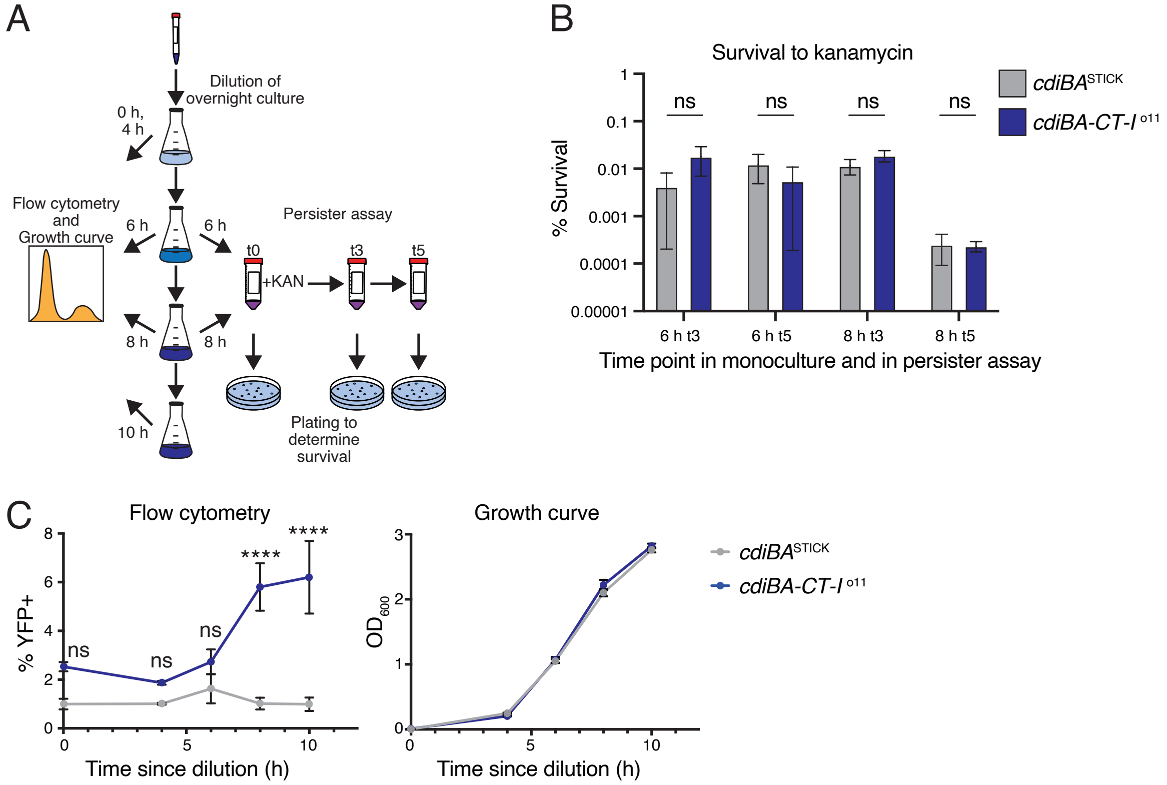

Figure S9. CdiA-CT^{o11} intoxication does not increase tolerance to Kanamycin. A) Overview of the experimental setup in B-C. **B)** *cdiBA-CT-I* ^{o11} and *cdiBA*^{STICK} cells from the monoculture were subjected to KAN treatment for 5 h. Viability was assessed using plating for cfu/mL. Survival 3 and 5 h post KAN treatment is shown. (N = 5 biological replicates). **C)** Time-resolved enumeration of growth (right) and YFP+ cells (left) in monocultures grown in M9-gly-CAA for 10 h. MG1655 with the *sulA*-sYFP2 reporter and *cdiBA-CT-I* ^{o11} (blue) or p*cdiBA*^{STICK} (grey) were used. (N = 5 biological replicates). Error bars are SEM. Statistical significance was determined through Student's t-test for B and two-way ANOVA with Bonferroni's posthoc test for C. Significance in C is relative to the *cdiBA*^{STICK} strain. * <0.05, ** <0.01, *** <0.001, ****<0.0001.

Supplementary tables

Table S1. Transcriptomics data. Raw data for differential mRNA levels (log2fold change) at 5 min (o11 5 min) and 20 min (o11 20 min) after induction of the pBAD33::*cdiA-CT* $^{\mathrm{o11}}$ vector. GoTerm analysis of the genes with statistically (P-value >0.01) changed expression 5min after intoxication (GoTerm analysis). Data is found in a separate excel file.

Table S2. Bacterial strains used in this study.

**Strain number**	**Genotype**	**Figure**	**Name in figure**	**Reference**
SK212	*E. coli* MG1655 *galK::BFP-FRT, sulA-YFP-cat* /pZS21::*bamA*^{Eco}	S5AB CD	p*bamA*	This study
SK213	*E. coli* MG1655 *lacA-cdiBA*$^{\mathrm{EC93\ -STICK}}$, *galK::cat*-pJ23101-*mTagBFP2*, *sulA-sYFP2-cat* /pZS21::*bamA*^{Eco}	4B	p*bamA cdiBA*^{STICK}	This study
SK214	*E. coli* MG1655 *lacA-cdiBA cdiBA*$^{\mathrm{EC93}}$*-CT-I* $^{\mathrm{o11}}$, *galK*:: pJ23101 *mTagBFP2*, *sulA-sYFP2-cat* /pZS21::*bamA*^{Eco}	4B	p*bamA cdiBA-CT-I* $^{\mathrm{o11}}$	This study
SK237	*E. coli* MG1655 *galK::BFP-FRT, sulA-YFP-cat* /pZS21::*bamA-yciB-kan*	S5AB CD	p*bamA yciB*	This study
SK238	*E. coli* MG1655 *lacA-cdiBA*(EC93), *galK::BFP-FRT, sulA-YFP-cat* /pZS21::*bamA-yciB-kan*	S5E	p*bamA yciB cdiBA*^{STICK}	This study
SK239	*E. coli* MG1655 *lacA-cdiBA*(EC93)-EC869 orphan 11 (CT+I), *galK::BFP-FRT, sulA-YFP-cat* /pZS21::*bamA-yciB-kan*	S5E	p*bamA yciB cdiBA-CT-I* $^{\mathrm{o11}}$	This study
SK284	*E. coli* MG1655 *galK::BFP-FRT, sulA-YFP-cat* /pZS21-MCS (*kan*)	S5AB CD	EVC	This study
SK285	*E. coli* MG1655 *lacA-cdiBA*(EC93), *galK::BFP-FRT, sulA-YFP-cat*, /pZS21-MCS (*kan*)	4BC, S5E	EVC *cdiBA*^{STICK}	This study
SK286	*E. coli* MG1655 *lacA-cdiBA*(EC93)-EC869 orphan 11 (CT+I), *galK::BFP-FRT, sulA-YFP-cat* /pZS21-MCS (*kan*)	4BC, S5E	EVC *cdiBA-CT-I* $^{\mathrm{o11}}$	This study
SK620	*E. coli* MG1655 *lacA-cat*			[5]
SK637	*E. coli* MG1655 *galK*::*cat*-J23101-*mTagBFP2*			[6]
SK806	*E. coli tisB-sYFP2-cat*			[7]
SK807	*E. coli sulA-sYFP2-cat*			[7]
SK844	*E. coli* MG1655 *galK*::*mTagBFP2*-FRT, *sulA-sYFP2-cat*	1	Background	This study
SK1344	*E. coli* MG1655 /pDAL930 (pDAL660 - EC869 orphan 11 chimera, *cat*)	S2A	pWEB::*cdiBA-CT-I* $^{\mathrm{o11}}$	
SK1371	*E. coli* MG1655 / pBAD33 empty	5AC S6AB		
SK2336	*E. coli sulA-sYFP2-kan*			This study
SK2528	*E. coli* MG1655, *bamA*$^{Sty\mathrm{LT2}}$ FRT-*kan*-FRT			[5]

SK2540	*E. coli* MG1655 *bamA*$^{Sty\mathrm{LT2}}$	S5BCD, S7EF		[5]
SK2827	*E. coli* MG1655 *lacA-kan*	S7C		[8]
SK2660	*E. coli* MG1655 *lacA-cdiBA*$^{\mathrm{EC93\ \text{-}STICK}}$*-kan*	S1CD, S2B	Chromosomal *cdiBA*^{STICK}	[5]
SK2708	*E. coli* MG1655 *lacA-cdiBA*$^{\mathrm{EC93}}$*-CT-I* $^{\mathrm{o11}}$*-kan*	S1CD	Chromosomal *cdiBA-CT-I* $^{\mathrm{o11}}$	This study
SK3801	*E. coli* MG1655 *galK::cat*-J23101-*dTomato*	S1CD		
SK4901	*E. coli* MG1655, *bamA*$^{\mathrm{StyLT2}}$ /pDAL930::EC93-EC869 o11 CT(D198A)-I	S8AB	p*cdiBA-CT-I* $^{\mathrm{o11(D198A)}}$	This study
SK4939	*E. coli* MG1655 *bamA*$^{Sty\mathrm{LT2}}$/pDAL930	3BC, 4A, S4, S5ABCD, S7EF	*cdiBA-CT-I* $^{\mathrm{o11}}$	This study
SK5074	*E. coli* MG1655 *lacA-cdiBA*$^{\mathrm{EC93\ \text{-}STICK}}$, *galK*::J23101-*mTagBFP2*, *tisB-sYFP2-cat*	S6D	*tisB-sYFP2* *cdiBA*^{STICK}	This study
SK5075	*E. coli* MG1655 *lacA-cdiBA*$^{\mathrm{EC93}}$*-CT-I* $^{\mathrm{o11}}$, *galK*::J23101-*mTagBFP2,tisB-sYFP2-cat*	S6D	*tisB-YFP* *cdiBA-CT-I* $^{\mathrm{o11}}$	This study
SK5076	*E. coli* MG1655 *lacA-cdiBA*$^{\mathrm{EC93\ \text{-}STICK}}$, *galK*::J23101-*mTagBFP2*, *sulA-sYFP2-cat*	1CDE, 4D , 5BC, S6D, S9	*cdiBA*^{STICK}	This study
SK5077	*E. coli* MG1655 *lacA-cdiBA*$^{\mathrm{EC93}}$*-CT-I* $^{\mathrm{o11}}$, *galK*::J23101-*mTagBFP2*, *sulA-sYFP2-cat*	1CDE, 4D, 5BC, S6D, S9	*cdiBA-CT-I* $^{\mathrm{o11}}$	This study
SK5123	*E. coli* MG1655 _*wzb* ara::*spec*/ pBAD33-*cdiI* $^{\mathrm{o11}}$-α-*mTagBFP2*, (5'UTR: AAAGAAATACTAG$^{(-1)}$)	2BC, S3CD	Target strain	This study
SK5351	*E. coli* MG1655 *galK*::*mTag-BFP2*-FRT, *sulA-sYFP2-kan*	1B, S1, S8AB	Target strain	This study
SK5373	*E. coli* MG1655, *galK*::J23101-*dTomato*	1CD, S1A	CDI-	[9]
SK5404	*E. coli* MG1655, *galK*::J23101-*dTomato* /pWEB-TNK	1B, S1, S6C	EVC	This study
SK5407	*E. coli* MG1655, *galK*::J23101-*dTomato* /pDAL930	1B, S1, S6C	*cdiBA-CT* $^{\mathrm{o11}}$	This study
SK5454	*E. coli* MG1655_*wzb ara::spec*/ pBAD33-*cdiI* $^{\mathrm{o11}}$-*mTagBFP2*	S3AB	N/A	This study
SK5831	*E. coli* MG1655 *galK*::*mTagBFP2*-FRT, *sulA-sYFP2-kan* /pBAD33^{weak}	3BC, 4A,	NC	This study

		S4, S8AB		
SK5836	*E. coli* MG1655 *galK*::*mTagBFP2*-FRT, *sulA*-SYFP2-*kan* /pBAD33 ^{weak}-*cdiI* ^{o11}	3BC, 4A, S4, S7EF	IndImm	This study
SK6346	*E. coli* MG1655 *bamA*^{StyLT2} /pWEB-TNK	4B	EVC	This study
SK6374	*E. coli* MG1655 *bamA*^{StyLT2} /pDAL660 CdiA-CT+I KO	S2AE	pWeb:: *cdiBA*^{STICK}	This study
SK6552	*E. coli* MG1655, *galK*::J23101-*dTomato* /pDAL878	1B, S1A	CDI-	This study
SK6599	*E. coli* MG1655 *lacA-cdiBA*(EC93), *galK::cat*-J23101-*dTomato*	S1CD	*cdiBA*^{STICK}	This study
SK6600	*E. coli* MG1655 *lacA-cdiBA*(EC93)-EC869 orphan 11 (CT+I), *galK::cat*-J23101-*dTomato*	S1CD	*cdiBA-CT-I* ^{o11}	This study
SK6626	*E. coli* MG1655, *galK*::J23101-*dTomato*, *bamA-LT2*-FRT	S8AB	CDI-	This study
SK6630	*E. coli* MG1655, *galK*::J23101-*dTomato*, *bamA-LT2*-FRT/ *pcdiBA-CT-I* ^{o11}	S8AB	p*cdiBA-CT-I* ^{o11}	[1]
SK6634	*E. coli* MG1655, *galK*::J23101-*dTomato*, *bamA-LT2*-FRT/ *pcdiBA-CT-I* ^{EC93}	S8AB	p*cdiBA-CT-I* ^{EC93}	[10]
SK6689	*E. coli* MG1655, *galK*::J23101-*dTomato*, *bamA-LT2*-FRT/*pcdiBA-CT-I* ^{UPEC536}	S8AB	p*cdiBA-CT-I* ^{UPEC536}	[11]
SK6732	*E. coli* MG1655 *lacA-cdiBA*$^{EC93\text{ -STICK}}$, *galK*::J23101-*mTagBFP2, sulA-sYFP2-kan*	S7C	*cdiBA*^{STICK}	This study
SK6733	*E. coli* MG1655 *lacA-cdiBA*^{EC93}-*CT-I* ^{o11}, *galK*::J23101-*mTagBFP2, sulA-sYFP2-kan*	S7C	*cdiBA-CT-I* ^{o11}	This study
SK6736	*E. coli* MG1655 *lacA-cdiBA*$^{EC93\text{ -STICK}}$, *galK*::J23101-*mTagBFP2*, *sulA-sYFP2-cat*, *bamA*$^{Sty\text{LT2}}$ FRT-*kan*-FRT	1E	*bamA*^{Sty} *cdiBA*^{STICK}	This study
SK6737	*E. coli* MG1655 *lacA-cdiBA*^{EC93}-*CT-I* ^{o11}, *galK*::J23101-*mTagBFP2*, *sulA-sYFP2-cat*, *bamA*$^{Sty\text{LT2}}$ FRT-*kan*-FRT	1E	*bamA*^{Sty} *cdiBA-CT*^{o11}	This study
SK6767	*E. coli* MG1655 *galK*::J23101-*mTagBFP2*, *sulA-sYFP2-kan*/ pBAD33::*CdiA-CT* ^{o11}(D198A)- α	S2C	*pBAD33::cdiA -CT*^{o11*}- α	This study
SK6768	*E. coli* MG1655 *galK*::J23101-*mTagBFP2*, *sulA-sYFP2-kan*/ pBAD33::*CdiA-CT* ^{o11}(D198A)	S2C	*pBAD33::cdiA -CT*^{o11*}	This study
SK6819	*E. coli* MG1655, *bamA*$^{Sty\text{LT2}}$ FRT.*kan*.FRT/ pDAL930- *cdiBA*^{EC93}-*CT*^{o11}-α-*I* ^{o11}-*HA*	2BC, S3CD	*cdiBA-CT* ^{o11}	This study
SK6861	*E. coli* MG1655 *lacA-cdiBA*^{EC93}-*CT-I*^{o11} */pBAD33(RBS/5'UTR-var2-o11-CT)*	5AC, S6AB	*cdiA-CT* ^{o11}	This study
SK7198	*E. coli* MG1655 *lacA-cdiBA*(EC93)-EC869 orphan 11 (CT+I), *galK::J23101-mTagBFP2, sulA-SYFP2-kan* /pBAD33(*cat*)	S1B	EVC	This study
SK7199	*E. coli* MG1655 *lacA-cdiBA*(EC93)-EC869 orphan 11 (CT+I), *galK::J23101-mTagBFP2, sulA-SYFP2-kan* /pBAD33(RBS/5'UTR-var2-o11-CT), *cat*	S1B	p*cdiA-CT* ^{o11}	This study

SK7200	*E. coli* MG1655 _*wzb ara::spec, galK::BFP-FRT, tisB-YFP-cat*	S6C	Target strain	This study
SK7813	*E. coli* DH5α/pZS21-*yciB*(variant: P114S)			This study
SK7818	*E. coli* MG1655 *lacA-cdiBA*(EC93), *galK::cat-J23101-mTagBFP2*, *sulA-SYFP2-cat* /pZS21-*yciB-kan*	4C	p*yciB* *cdiBA* ^{STICK}	This study
SK7819	*E. coli* MG1655 *lacA-cdiBA*(EC93)-EC869 orphan 11 (CT+I), *galK::J23101-mTagBFP2*, *sulA-SYFP2-cat* /pZS21-*yciB-kan*	4C	p*yciB cdiBA-CT* ^{o11}	This study
SK7820	*E. coli ompC::tet.*			This study
SK7823	*E. coli* MG1655 *lacA-cdiBA*(EC93), *galK::cat-J23101-mTagBFP2*, *sulA-SYFP2-cat*, *ompC::tet*	4D	Δ*ompC* *cdiBA* ^{STICK}	This study
SK7824	*E. coli* MG1655 *lacA-cdiBA*(EC93)-EC869 orphan 11 (CT+I), *galK*::J23101-*mTagBFP2*, *sulA-SYFP2-cat*, *ompC::tet*	4D	Δ*ompC* *cdiBA* ^{STICK}	This study

Table S3. Plasmids used in this study

Plasmid	Plasmid description	Resistance on plasmid	Name	Reference
pWEB-TNC	Empty vector for pDAL878 and pDAL930	AMP CAM	EVC	GenBank: EU140755.1
pSK479	pWEB::*cdiBA-CT-I* ^{UPEC536}	AMP	p*cdiBA-CT-I* UPEC536	[11]
pDAL660Δ1-39	pWEB::*cdiBA-CT-I* ^{EC93}	AMP	p*cdiBA-CT-I* *EC93*	[10]
pDAL930	pWEB::*cdiBA-CT-I* ^{o11}	AMP	p*cdiBA-CT-I* ^{o11}	[1]
pCH9305	pWEB::*cdiBA-CT-I* ^{o11}	CAM	p*cdiBA-CT* ^{o11}	[12]
pSK4896	pWEB::*cdiBA-CT* $^{(D198A)}$*-I* ^{o11}	CAM	p*cdiBA-CT* ^{o11*}	This study
pCH10163	pWEB::*cdiBA-pheS-kan*	AMP KAN		[1]
pSK5111	pBAD33 *cdiI* ^{o11}-*mTagBFP2* (5'UTR: AGAGAAATACTAG$^{(-1)}$)	CAM	*cdiI* ^{o11}-*mTagBFP2*	This study
pSK5115	pBAD33 *cdiI* ^{o11}-α-*mTagBFP2*, (5'UTR: AAAGAAATACTAG$^{(-1)}$)	CAM	*cdiI* ^{o11}-*α*	This study
pSK5244	pBAD33^{medium}	CAM	pBAD^{medium}	This study
pSK5248	pBAD33 ^{weak}	CAM	pBAD^{weak}	This study
pSK5346	pBAD33^{weak}-*cdiI* ^{o11} (5'UTR:GAGCTCGGTACC$^{(-1)}$)	CAM	pBAD^{weakRBS}-*cdiI* ^{o11}	This study
pDAL878	pWEB:: *cdiBA*$^{EC93\ -STICK}$	AMP	*cdiBA*^{STICK}	[13]
pSK6819	pWEB::*cdiBA-CT* ^{o11}-α-*I* ^{o11}-*HA*	CAM	*cdiBA-CT* ^{o11}-α, *cdiI* ^{o11}-*HA*	This study

Table S4. Oligos used in this study

Oligo nr	Sequence	Description
345	GATATACCGGACATCCGCAACAGC	Verification of inserting *cdiBA*^{STICK} on the chromosome at *lacA*
346	GCTGTTATCCAGTGAGTTTCCATCAAG	Verification of inserting *cdiBA*^{STICK} on the chromosome at *lacA*
347	GTTCGGATGTGGTGGCTGACC	Verification of inserting *cdiBA*^{STICK} on the chromosome at *lacA*
348	GTCCACAGTACGACGGTCATGC	Verification of inserting *cdiBA*^{STICK} on the chromosome at *lacA*
349	GAAGGCAGGCTGCAGCAAATCC	Verification of inserting *cdiBA*^{STICK} on the chromosome at *lacA*
350	ACCGAAAAGTCACTGCTCCGTCC	Verification of inserting *cdiBA*^{STICK} on the chromosome at *lacA*
387	CGCTTCAGCCATACTTTTCA	pBAD verification primer
388	GTCTCATGAGCGGATACATATTTG	pBAD verification primer
690	GTTGGTAGTGGTGGTGCTG	pDAL_down_rev
1083	CGCGCCTTACGCCCCGCCCTGCCACTCA GAAGAACTCGTCAAGAAGGC	Insertion of *sYFP2-kan* down-stream of *sulA*.
1785	TGTAGATCTTTAACCTTTGCAGCGAC	Cloning of *cdiI*^{o11}. Contains *Bgl*II site
1778	TGTAGATCTAGGAGAAATACTAGATGA GCGAACTGATCAAA	Cloning of *mTagBFP*. Contains *Bgl*II site
1779	TGTAAGCTTTTAATTCAGTTTATGACCC AG	Cloning of *mTagBFP*. Contains *Hind*III site
2071	ATGGCTCATGCAGTCTGG	Insertion of *cdiBA-CT*^{o11}-*I*^{o11} on the stick
2074	CCCCAGATCTTGGACAACCATATGAATA TCCTCCTTAGTTCC	Insertion of *cdiBA-CT*^{o11}-*I*^{o11} on the stick
2075	GGAACTAAGGAGGATATTCATATGGTT GTCCAAGATCTGGGG	Insertion of *cdiBA-CT*^{o11}-*I*^{o11} on the stick
2076	CTC AAC GAC AGG AGC ACG	Insertion of *cdiBA-CT*^{o11}-*I*^{o11} on the stick
2077	GCAGCTCCAGCCTACACTTAACCTTTGC AGCGACTCAAG	Insertion of *cdiBA-CT*^{o11}-*I*^{o11} on the stick
2078	CTT GAG TCG CTG CAA AGG TTA AGTGTAGGCTGGAGCTGC	Insertion of *cdiBA-CT*^{o11}-*I*^{o11} on the stick
2100	GTATGTCCGGCAGCAGTG	Sequencing primer to verify insertion of *cdiBA-CT*^{o11}-*I*^{o11} on the stick
2225	ATATTCTAGAAGCATTTGATTACGGAAT CGTA	Cloning of *yciB*. Contains *Xba*I site
2226	GCGCGTCGACGGCAGTCTGGTTAGGATT TATC	Cloning of *yciB*. Contains *Sal*I site
2231	CCGTTGGTGTTCTCCTACG	Verification of pZS21 plasmids forward
2232	TCATGGCAATTCTGGAAGAA	Verification of pZS21 plasmids reverse

2593	TCTCAGGTACCATGGCTTTTAATAAAGATCAGG	Cloning of *cdiI* ^{o11}, contains a *Kpn*I site
2594	TCTCAAAGCTTTTAACCTTTGCAGCGACTC	Cloning of *cdiI* ^{o11}, contains a *HindIII* site
2777	TCTCTGGTACCCGGGTCTAGAGTCGACAAGCTTGGCTGTTTTGG	Cloning of pBAD33 with weak RBS. Contains a *Kpn*I site
2778	TCTCTGGTACCGAGCTCGAATTCG	Cloning of pBAD33 with weak RBS. Contains a *Kpn*I site
2781	ACAGAATTCAAAGAAATACTAGATGGCTTTTAATAAAGATCAGG	Cloning of *cdiI* ^{o11}. Contains an *Eco*RI site
2782	TATAGATCTTTATTCGGTCAGGCGGCGGCGCAGTTCTTCTTCCAGGCGGCTACCTTTGCAGCGACTCAAG	Cloning of *cdiI* ^{o11}-α. Contains an *Bgl*II site
2783	CTCATCTGAATTCAGAGAAATACTAGATGGC	Primer to change RBS in pBAD33 to RBS(-G). Contains an *Eco*RI site
2784	CAGGAACGTCAGCAAATCAG	Seq_pCH7876_forward
2785	AGCCGCCTGGAAGAAGAACTGCGCCGCCGCCTGACCGAGTAAACGCAGGTGAAATAATGGCTTTTAAT	Insertion of *cdiI* ^{o11}-α on the chromosome and in pDAL930
2786	CTCGGTCAGGCGGCGGCGCAGTTCTTCTTCCAGGCGGCTTTTCACCTGCGTTACCGTCAC	Insertion of *cdiI* ^{o11}-α on the chromosome and in pDAL930
2791	CCGTATGATGTGCCGGATTATGCGTAACCCAAAGGTTAGACACCAG	Insertion of *cdiI* ^{o11}-HA in pDAL930
2792	ATAATCCGGCACATCATACGGATAACCTTTGCAGCGACTCAAG	Insertion of *cdiI* ^{o11}-HA in pDAL930
2793	GAACTTTAAGATCTTTGCGTACTATGATGGTGC	Construction of *cdiA-CT* ^{o11}(H198A) mutation on pDAL930
2794	ATCATAGTACGCAAAGATCTTAAAGTTCTTTGG	Construction of *cdiA-CT* ^{o11}(H198A) mutation on pDAL930
2796	TCTCTGGTACCTTTCTTTAATTCGCTAGCCCAAAAAAAC	Cloning of pBAD33 with medium RBS. Contains a *Kpn*I site

Supplementary references:

1. Morse, R.P., K.C. Nikolakakis, J.L. Willett, E. Gerrick, D.A. Low, C.S. Hayes, and C.W. Goulding, *Structural basis of toxicity and immunity in contact-dependent growth inhibition (CDI) systems.* Proc Natl Acad Sci U S A, 2012. **109**(52): p. 21480-5.
2. Datsenko, K.A. and B.L. Wanner, *One-step inactivation of chromosomal genes in Escherichia coli K-12 using PCR products.* Proc Natl Acad Sci U S A, 2000. **97**(12): p. 6640-5.
3. Lennox, E.S., *Transduction of linked genetic characters of the host by bacteriophage P1.* Virology, 1955. **1**(2): p. 190-206.
4. Kim, S., J.C. Malinverni, P. Sliz, T.J. Silhavy, S.C. Harrison, and D. Kahne, *Structure and function of an essential component of the outer membrane protein assembly machine.* Science, 2007. **317**(5840): p. 961-4.
5. Ghosh, A., O. Baltekin, M. Waneskog, D. Elkhalifa, D.L. Hammarlof, J. Elf, and S. Koskiniemi, *Contact-dependent growth inhibition induces high levels of antibiotic-tolerant persister cells in clonal bacterial populations.* EMBO J, 2018. **37**(9).
6. Gullberg, E., S. Cao, O.G. Berg, C. Ilback, L. Sandegren, D. Hughes, and D.I. Andersson, *Selection of resistant bacteria at very low antibiotic concentrations.* PLoS Pathog, 2011. **7**(7): p. e1002158.
7. Berghoff, B.A., M. Hoekzema, L. Aulbach, and E.G. Wagner, *Two regulatory RNA elements affect TisB-dependent depolarization and persister formation.* Mol Microbiol, 2017. **103**(6): p. 1020-1033.
8. Virtanen, P., M. Waneskog, and S. Koskiniemi, *Class II contact-dependent growth inhibition (CDI) systems allow for broad-range cross-species toxin delivery within the Enterobacteriaceae family.* Mol Microbiol, 2019. **111**(4): p. 1109-1125.
9. Gullberg, E., L.M. Albrecht, C. Karlsson, L. Sandegren, and D.I. Andersson, *Selection of a multidrug resistance plasmid by sublethal levels of antibiotics and heavy metals.* mBio, 2014. **5**(5): p. e01918-14.
10. Aoki, S.K., R. Pamma, A.D. Hernday, J.E. Bickham, B.A. Braaten, and D.A. Low, *Contact-dependent inhibition of growth in Escherichia coli.* Science, 2005. **309**(5738): p. 1245-8.
11. Aoki, S.K., E.J. Diner, C.T. de Roodenbeke, B.R. Burgess, S.J. Poole, B.A. Braaten, A.M. Jones, J.S. Webb, C.S. Hayes, P.A. Cotter, and D.A. Low, *A widespread family of polymorphic contact-dependent toxin delivery systems in bacteria.* Nature, 2010. **468**(7322): p. 439-42.
12. Willett, J.L., G.C. Gucinski, J.P. Fatherree, D.A. Low, and C.S. Hayes, *Contact-dependent growth inhibition toxins exploit multiple independent cell-entry pathways.* Proc Natl Acad Sci U S A, 2015. **112**(36): p. 11341-6.
13. Webb, J.S., K.C. Nikolakakis, J.L. Willett, S.K. Aoki, C.S. Hayes, and D.A. Low, *Delivery of CdiA nuclease toxins into target cells during contact-dependent growth inhibition.* PLoS One, 2013. **8**(2): p. e57609.
